# An organoid library of salivary gland tumors reveals subtype-specific characteristics and biomarkers

**DOI:** 10.1186/s13046-022-02561-5

**Published:** 2022-12-17

**Authors:** Bo Wang, Jiaxing Gan, Zhengyan Liu, Zhixuan Hui, Jinhui Wei, Xiaolian Gu, Yabing Mu, Guangxiang Zang

**Affiliations:** 1 Liaoning Provincial Key Laboratory of Oral Disease, School and Hospital of Stomatology, China Medical University, Nanjingbeijie 117, Shenyang City, 110051 People’s Republic of China; 2 Department of Medical Bioscience, Building 6M, Umeå University, 90185 Umeå, SE Sweden

**Keywords:** Salivary gland tumors, Organoids, Cluster analysis, Biomarkers

## Abstract

**Background:**

Salivary gland tumors (SGTs) include a large group of rare neoplasms in the head and neck region, and the heterogeneous and overlapping features among the subtypes frequently make diagnostic difficulties. There is an urgent need to understand the cellular mechanisms underlying the heterogeneity and overlap among the subtypes, and explore the subtype-specific diagnostic biomarkers.

**Methods:**

The tumor tissue and the adjacent normal tissue from the 6 most common types of SGTs were processed for organoid culture which only maintained tumor epithelial cells. Organoids were histologically evaluated based on phenotype markers, followed by transcriptional profiling using RNA-sequencing. The transcriptomic similarities and differences among the subtypes were analyzed by subtype consensus clustering and hierarchical clustering. Furthermore, by comparative transcriptional analysis for these 6 types of SGTs and the matched organoids, the potential diagnostic biomarkers from tumor epithelium were identified, in which two selected biomarkers were evaluated by qPCR and confirmed by immunohistochemistry staining using a tissue microarray.

**Results:**

We generated a biobank of patient-derived organoids (PDOs) with 6 subtypes of SGTs, including 21 benign and 24 malignant SGTs. The PDOs recapitulated the morphological and transcriptional characteristics of the parental tumors. The overlap in the cell types and the heterogenous growth patterns were observed in the different subtypes of organoids. Comparing the bulk tissues, the cluster analysis of the PDOs remarkably revealed the epithelial characteristics, and visualized the intrinsic relationship among these subtypes. Finally, the exclusive biomarkers for the 6 most common types of SGTs were uncovered by comparative analysis, and PTP4A1 was demonstrated as a useful diagnostic biomarker for mucoepidermoid carcinoma.

**Conclusions:**

We established the first organoid biobank with multiple subtypes of SGTs. PDOs of SGTs recapitulate the morphological and transcriptional characteristics of the original tumors, which uncovers subtype-specific biomarkers and reveals the molecular distance among the subtype of SGTs.

**Supplementary Information:**

The online version contains supplementary material available at 10.1186/s13046-022-02561-5.

## Background

SGTs are one of the most challenging areas for diagnostic pathology, with a lower incidence of about 0.5–2 cases per 100.000 yearly [1–3], but a large number of subgroups for pathological classification, including 11 types of benign and 20 types of malignant entities of epithelial origin [4]. These subtypes often display similar aberrant and pleomorphic structures, such as solid, trabecular, tubular, glandular and ductal patterns, papillary projections, cystic space, and so on. These complex pathological morphologies with widely heterogeneous and overlapping features among subgroups, make challenges for the pathological diagnosis [5].

The normal salivary gland (NSG) has regular patterns and structures, consisting of acini and the duct system. The acini are composed of acinus cells surrounded by myoepithelial cells, and the duct system consists of intercalated ducts, striated ducts, and excretory ducts. It is generally accepted that there are basal stem cells in excretory ducts and multipotent stem cells residing in the intercalated ducts, important for maintaining NSG homeostasis. However, these stem cells are also suspected as the origin of SGTs [6].

Although recent studies tried to characterize the molecular profile of SGTs, most studies only focus on certain subtypes [7–11]. Since the overlap in morphologies and cell types are common among the subgroups, it is difficult to know if this molecular profile is unique for one subtype, or has overlapped with the other subtypes. In fact, due to the limited number of rare SGTs in clinics and the lack of research models to study these SGTs, the comparative molecular profiles among SGTs subtypes were rarely characterized.

In the past few years, three-dimensional (3D) culture technologies have been rapidly developed, and culturing PDOs has become a powerful clinical translational research model. Tumor organoids derived from patients could preserve the morphologic and genetic characteristics of the original tumors, and were broadly utilized for drug screening and biomarker identification [12–14]. In addition, PDOs could enable the survival or outgrowth of epithelial cells in vitro in both short-term and long-term, functioning as a practical model to study rare diseases [15, 16]. Importantly, the organoids model lacks human stroma and defined less complex molecular subgroups than the entire tissue, thus becoming a practical model to characterize the complicated tumor subtypes [17, 18].

Herein, we established a biobank with 45 PDOs of SGTs obtained from surgical resection, including 11 pleomorphic adenoma (PA), 10 basal cell adenoma (BCA), 10 adenoid cystic carcinoma (ACC), 6 mucoepidermoid carcinoma (MEC), 4 acinic cell carcinoma (AciCC), 4 salivary duct carcinoma (SDC). Transcriptome-based clustering and comparisons among PDOs and original tumor tissues demonstrate the molecular distance between subgroups and the utility of PDOs in discovering diagnostic biomarkers.

## Material and methods

### Collection of patient samples

SGTs tissue and the marched adjacent NSG were taken from surgically resected specimens in Stomatology Hospital, China Medical University (Shenyang, China). The clinical research protocol was approved by the ethics committee (2020–03, Stomatology Hospital, China Medical University), and was performed following the Declaration of Helsinki and good clinical practice guidelines. Diagnosis of the tumors was confirmed by two pathologists in the hospital.

### Tissue preparation and organoid establishment

After surgery, patient tissues were collected and kept in cold PBS with Penicillin, streptomycin, and amphotericin (Gibco), then trimmed into 1–3 mm^3^ pieces without connected tissues and blood. Tissue pieces were preserved in the liquid nitrogen for RNA isolation, while the tissue was saved for organoid culture following the routine protocols [19, 20]. Briefly, tissue pieces were incubated with 1x Dispase (Roche, IN, USA) at 37 °C for a maximum of 60 minutes with gentle agitation. After dissociation, the cell suspension was centrifuged at 800 rpm/min for 3 min. The pellets were resuspended in ad-DMEM/F12 medium (Gibco), passed through 100-μm cell strainers (BD Falcon), and then centrifuged at 1000 rpm for 5 min. The resulting pellet was embedded in Matrigel (Corning) and seeded in 24-well culture plates with 40 μl Matrigel each well. After solidifying for 30 min at 37 °C, 600 μl organoid medium was added to each well and cultured at 37 °C, 5% CO_2,_ and humidity environment. The culture media for tumor organoid was same as Driehuis’ protocol [21], ad-DMEM/F12, supplemented with GlutaMAX, 1% penicillin/streptomycin (Gibco), B27 (Invitrogen), 1.25 mM N-acetyl-L-cysteine, 3 μM CHIR99021, 50 ng/ml Noggin, 100 ng/ml R-Spondin-1, 50 ng/ml FGF-10 (Invitrogen, CA, USA), 50 ng/ml human EGF (Invitrogen), 0.1 μM A83–01, 10 μM Y27632, and 1 μM Dexamethasone. The culture media for NSG organoids was same as Yoshimoto’s protocol [22], an additional 20 ng/ml FGF-2, Insulin-Transferrin-Selenium, and 0.05 mg/ml Heparin were added. The medium was changed every 2–3 days, and the organoids were passaged after 1–3 weeks. Mature cultured organoids were cryopreserved in a cryopreservation medium (FBS: DMSO = 9:1) and stored in liquid nitrogen after gradient cooling. Organoid cultures were tested routinely for Mycoplasma by PCR in the laboratory.

### H&E, IHC, and immunofluorescence (IF) staining

The histology staining protocol followed the methods in the previous study [23]. Briefly, organoids were washed twice with cold PBS, fixed with 4% paraformaldehyde for 15 minutes, and then removed into the mold to dehydrate and embedded in paraffin. Sections were subjected to standard H&E, IHC, and IF staining. The following primary and secondary antibodies were used in this study: Cytokeratin 5 (CK5,1:200, EP1601Y, Invitrogen), Cytokeratin 8 (CK8, 1:200, sc-8020, Santa cruz), p63 (1:200, ab735, Abcam), Aquaporin 5 (AQP5, 1:400, ab92320, Abcam), alpha-smooth muscle actin (SMA,1:200, ab7817, Abcam), DOG-1 (GT205402, GeneTech), SOX-10 (GT221002, GeneTech), C-erbB-2 (HER-2, GT224502, GeneTech), Androgen receptor (AR, GM356202, GeneTech), MUC-4 (GT227502, GeneTech), PTP4A1(1:400, DF12458, Affinity), NEFL (1:50, ab223343, Abcam), Alexa Fluor 488 (1:300, A-21206, Invitrogen), Alexa Fluor 555 (1:300, A-31570, Invitrogen). Images were acquired with Olympus BX35 microscope and Olympus laser confocal microscope FV3000.

### AB-PAS staining

Following the protocol of the AB-PAS kit (BA4121, BASO), paraffin sections were routinely deparaffinized, washed with distilled water, and stained with Alcian staining solution (PH2.5) for 10 min. And then the sections were oxidized with periodate acid solution for 10 min, stained with schiff reagent for 10 min, and finally stained with Mayer for nuclei for 1 min. The acidic mucus was shown blue, the neutral mucus was red, and the mixed mucinous structure was shown purple-blue.

### Preparation of tissue microarray (TMA)

Paraffin-embedded tumor tissues of SGTs were collected and made into multiple tissue microarray blocks, following the permission of the ethics (2020–03, Stomatology Hospital, China Medical University). Briefly, the representative cores in 1.5 mm diameter from each tumor were drilled and collected by extracting cylindrical tissue cores from different donor blocks, and then were re-embedded into the recipient block in a defined pattern. TMA sections were used for the subsequent experiments.

### RNA extraction and sequencing

Organoids were collected from Matrigel. Total RNA from homogenized tumor tissue and pelleted organoids were kept in TRIzol (Invitrogen) and followed the standard protocol (BGI technology, China). The quality of the RNA samples was assessed by an Agilent Bioanalyzer (Agilent), and cDNA libraries were generated using TruSeq RNA Sample Preparation (Illumina). Each library was sequenced using single-reads on a HiSeq2000/1000 (Illumina).

### Quantitative real time-PCR (qPCR)

Reverse transcription of 1 μg RNA was performed using Hifair Strand cDNA Synthesis SuperMix (11123ES60, YEASEN). Quantification of mRNA expression was performed using Hieff qPCR SYBR Green Master (Low ROX, 11202ES08, YEASEN) and the cycle conditions were: 95 °C for 5 min and followed by 35 amplification cycles (95 °C for 10 seconds, 60 °C for 30 seconds). The results were represented as fold change using the ΔΔCt method. Primers used for qPCR included NEFL F 5′-TACAGACCAGCTCCTATCTGAT -3′, NEFL R 5’AATGGTTTCCTCCACTTCGATC -3′, PTP4A1 F 5’GTATCCATGTTCTTGATTGGCC-3′, PTP4A1 R 5’ACCAGGTTCTTCACGAAACTTA-3′, GAPDH F 5′- GACAGTCAGCCGCATCTTCT -3′, GAPDH R 5′- TTAAAAGCAGCCCTGGTGAC − 3.

### Dimensionality reduction to characterize subtypes of SGTs

Dimensionality reduction of the RNA-seq data was performed with the t-stochastic neighboring embedding (tSNE) method. Principal components of the transtSNE were set by R package Seurat (npcs = 20, perplexity = 11), which kept the distribution of each sample unchanged when the mapping was performed from high dimension to low dimension. The sample distribution in high dimensions was regarded as a gaussian distribution, while in low dimensions was regarded as T distribution [24].

### Subtype consensus clustering and hierarchical clustering

We performed k-means consensus clustering by using 5000 genes with the highest standard deviation (SD), and 500 repetitions to clarify the possible subtypes in SGTs and the matched organoids respectively with R package CancerSubtypes (ver.1.18.0) [25]. The number of K varies from 2 to 10 clusters, and was determined by the corresponding empirical cumulative distribution (cumulative distribution function plot). The number of clusters is decided when any further increase in cluster number (k) does not lead to a corresponding marked increase in the CDF area.

All SGTs and the matched organoids were identified by using hierarchical clustering analysis based on Euclidean distance and complete-linkage with R package hclust, and the outcome was transformed into tree plot by R package ggtree.V3.0.4 [26].

### Comparative transcriptional analysis for biomarker selection

With RNA-seq data, the original SGTs tissue was compared with NSG tissues, and PDOs were compared with NSG tissues respectively to obtain the differentially expressed genes (DEGs). Analysis of DEGs was performed by the DESeq2 with R package DESeq2. The intersection of DEGs from SGTs tissue and PDOs was further used to characterize the epithelial genes in SGTs. Lasso regression analysis was performed to obtain the exclusive epithelial genes in different types of SGTs via R package Glmnet (family = binomial, measure = deviance).

### Statistical analysis

Statistical analysis was performed with one-way ANOVA, *P* < 0.05 was marked as *, *P* < 0.01 as **, *P* < 0.001 as ***, *P* < 0.0001 as ****, while no significant difference was marked as n.s.

## Results

### Establishment of PDOs library of SGTs

**Fig. 1:**
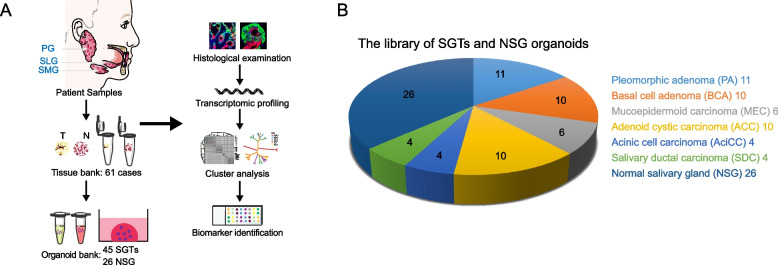
Establishment of PDOs library of SGTs. A A workflow for this study. B Pie chart showed the subtypes of SGTs and NSG organoids in the library

The workflow for this study was illustrated in Fig. 1 A. Totally, 8 categories of SGTs and the adjacent normal tissue, and the adjacent normal tissue from submandibular gland cyst (SGC) were obtained from 61 patients (Table 1). Following the protocols, we established a library with 45 PDOs that included 21 benign SGTs (PA: 11/13, BCA: 10/10) and 24 malignant tumors (MEC: 6/8, ACC: 10/12, AciCC: 4/4, SDC: 4/5) (Fig. 1 B). In addition, we cultured the matched normal organoids (26 out of 33) from the adjacent normal tissue (Table 1). Overall, the success rate of organoids cultures was 71/85 (84%). Those samples failed to set up the organoid models might due to the variation in tumor stroma. Typically, the unsuccessful cases contained a high percentage of tumor stroma in various morphology, such as the hyaline stroma in ACC, or the chondroid and osteoid stromal components in PA, which had fewer tumor cells than the others (Additional file 1 Supplementary Fig. S1A). Unfortunately, under the current culture system, the Warthin tumor, the second most common benign tumor in salivary glands (SGs) with dense lymphoid stroma, and oncocytoma, a rare benign tumor, could not initiate tumor organoids (Additional file 1 Supplementary Fig. S1B and C), and these cases were excluded from our final count. All the clinical characteristics for those tumors applied in this research were shown in Table 1.

**Table 1:** The list of the patient biobank

					Tissue			3D Organoids				Subtype
ID	Sex	Age	Tumor source	TNM	Type	Normal	Tumor	Normal	Passage	Tumor	Passage	Differentiation
1	M	41	SMG		PA	+	+	+	4	+	5	
2	F	32	SMG		PA	+	+	+	3	+	4	
3	M	55	SMG		PA	+	+	+	5	+	4	
4	M	63	PG		PA		+					
5	F	31	SMG		PA	+		+	4	+	5	
6	M	40	PG		PA	+	+	+	5	+	3	
7	F	43	PG		PA		+					
8	F	57	SMG		PA	+	+	+	5	+	5	
9	F	43	PG		PA	+	+	+	4	+	6	
10	F	64	SMG		PA	+	+	+	4	+	5	
11	F	40	PG		PA	+	+	+	5	+	5	
12	M	20	SMG		PA	+	+	+	5	+	4	
13	M	83	PG		PA	+		+	5	+	5	
14	F	44	PG		BCA	+	+			+	6	
15	F	66	PG		BCA	+	+	+	5	+	5	
16	M	74	PG		BCA	+	+	+	4	+	6	
17	M	56	PG		BCA					+	5	
18	F	64	PG		BCA	+	+	+	3	+	4	
19	F	61	PG		BCA	+	+			+	5	
20	F	59	PG		BCA	+	+	+	5	+	5	
21	F	47	PG		BCA	+	+	+	2	+	5	
22	F	56	PG		BCA	+		+	5	+	5	
23	F	59	PG		BCA	+	+			+	4	
24	M	23	PG	T2N0M0	MEC	+	+			+	5	Moderate
25	F	35	PG	T2N0M0	MEC		+					Moderate
26	F	53	MSG	T1N0M0	MEC		+					Moderate
27	M	33	PG	T1N0M0	MEC		+			+	4	Moderate
28	M	62	PG	T2N0M0	MEC	+	+			+	4	Well
29	M	46	PG	T2N0M0	MEC		+			+	3	Well
30	M	31	PG	T2N0M0	MEC	+	+	+	3	+	5	Well
31	F	62	SMG	T1N0M0	MEC	+	+			+	5	Moderate
32	F	79	SMG	T2N0M0	ACC		+			+	5	Tubular cribriform
33	F	54	SMG	T2N0M0	ACC	+	+			+	5	Cribriform tubuular
34	M	66	MSG	T1N0M0	ACC		+			+	3	Cribriform
35	F	43	SMG	T1N0M0	ACC		+			+	6	Tubular solid cribriform
36	F	60	SMG	T1N0M0	ACC		+					Cribriform tubular
37	M	48	MSG	T1N0M0	ACC		+			+	5	Tubular
38	F	60	SLG	T2N0M0	ACC		+					Tubular,cribriform
39	F	57	SMG	T1N0M0	ACC		+			+	5	cribriforf tubuular
40	F	47	MSG	T2N0M0	ACC	+	+		4	+	4	Cribriform
41	F	56	MSG	T2N0M0	ACC		+			+	6	Tubular cribriform
42	M	55	MSG	T2N0M0	ACC		+			+	5	Tubular cribriform
43	F	35	MSG	T2N0M0	ACC					+	5	Tubular cribriform
44	F	34	PG	T2N0M0	AciCC	+	+	+	5	+	3	Acinar
45	F	53	PG	T2N0M0	AciCC	+	+			+	2	Acinar
46	F	25	PG	T2N0M0	AciCC		+	+	5	+	3	Acinar
47	M	29	PG	T2N0M0	AciCC		+			+	2	Acinar
48	M	58	PG	T2N0M0	SDC	+	+	+	3			
49	M	65	SMG	T4N0M0	SDC	+	+	+	5	+	5	
50	F	43	SMG	T4N0M0	SDC	+	+	+	4	+	4	
51	M	53	PG	T4N0M0	SDC	+	+	+	5	+	5	
52	M	39	PG	T2N0M0	SDC	+	+	+	4	+	5	
53	M	47	PG		ME		+					
54	F	65	MSG		ME		+					
55	M	10	MSG		ME		+					
56	M	58	PG	T2N0M0	MC	+	+					Clear cell type
57	F	16	SLG		SGC	+						
58	F	48	SLG		SGC	+						
59	F	10	SLG		SGC	+						
60	F	32	SLG		SGC	+						
61	M	48	SLG		SGC	+						

Abbreviations: F, female; M, male; PG, parotid gland; SMG, submandibular gland; SLG, sublingual gland; MSG, minor salivary glands; PA, pleomorphic adenoma; BCA,basal cell adenoma; MEC,mucoepidermoid carcinoma; ACC,adenoid cystic carcinoma; Acicc, acinic cell carcinoma; SDC, salivary ductal carcinoma; ME, myoepithelioma; MC, myoepithelial carcinoma; SGC, submandibular gland cyst. +,successfully collected

**Fig. 2:**
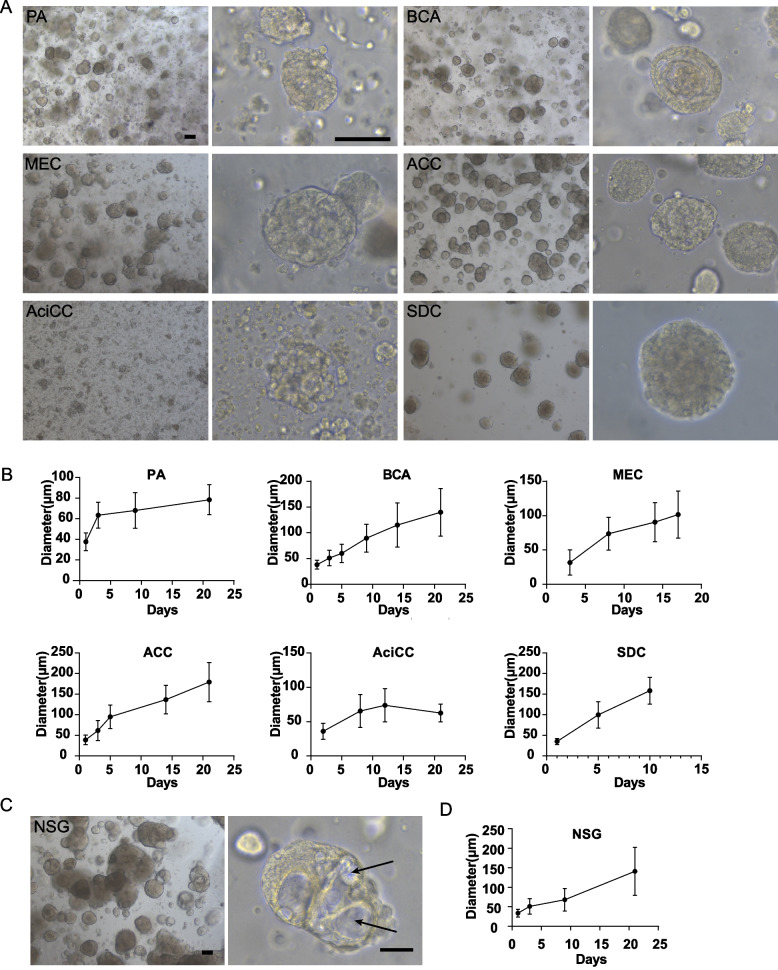
Structure and growth characteristics of PDOs. A Representative bright-field images of SGTs organoids. Scale bar, 100 μm. B Growth kinetics were analyzed by quantifying the average size (μm, mean ± S.D.) of 50 organoids from three independent samples. C Representative bright-field images of NSG, and the arrow pointed to the mucus secreted by NSG organoids. Scale bar, 100 μm. D Growth kinetics of NSG organoids from three independent samples

The initial stage of SGTs organoids was a process of self-assembly from single cells, and the expansion rates varied according to the tumor types (Fig. 2 A). Most types of tumors were in a rapid growth phase in the first week, could be firstly passaged approximately in 14 days, and then reached a plateau phase till 21 days (Fig. 2 B and Additional file 1 Supplementary Fig. S2), which was consistent with the previous report for NSG organoids [27]. Most organoids were expanded for five passages except for AciCC which has a lower proliferation rate (Table 1). Notably, we observed that NSG organoids were larger than SGTs, and the secreted mucus often appeared in the middle of NSG organoids (Fig. 2 C), which indicates that NSG organoids are functional. Interestingly, tumor organoids did not grow faster than the matched normal organoid counterparts, and in many cases even grew more slowly (Fig. 2 B and D). As reported in the other research, this might due to the higher rates of mitotic failures and subsequent cell death of tumor cells [27, 28].

### PDOs recapitulate the morphologic features of parental SGTs

**Fig. 3:**
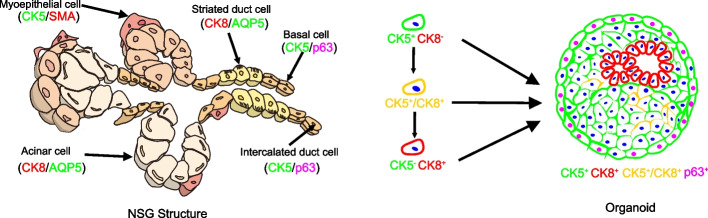
Schematic diagram showed the cell types and the biomarkers observed in this study

**Fig. 4:**
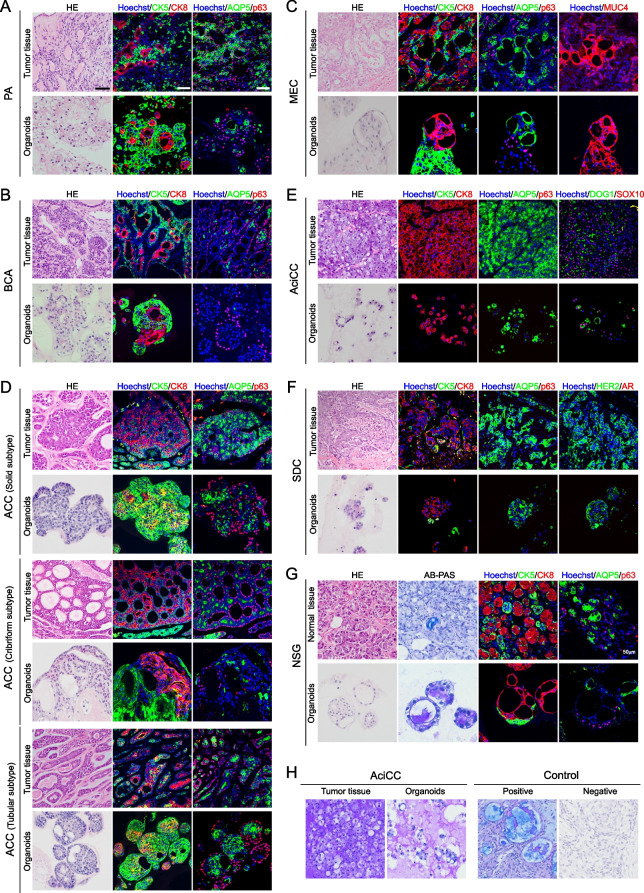
PDOs recapitulate the morphologic features of parental SGTs. Representative H&E images and confocal images of IF staining with the original tissues and the corresponding organoids. Two benign tumors PA (A), BCA (B); four malignant tumors MEC (C), ACC (D), AciCC (E), SDC (F), and NSG (G); AB-PAS staining of secreted mucus in the NSG (G) and AciCC (H). Clinical diagnostic biomarkers were used to evaluate the morphological and molecular features, including Cytokeratin 5 (CK5), Cytokeratin 8 (CK8), Aquaporin 5 (AQP5), p63, C-erbB-2 (HER2), Androgen Receptor (AR), MUC4, DOG1, SOX10. Scale bar, 50 μm

Current classifications of SGTs depends on histological morphology and a few biomarkers. Under the brightfield microscope, all types of organoids showed mainly ball-like structures and irregular tumor mass shapes in the culture plate (Fig. 2). To compare the morphological and histological features of PDOs with the parental tissues, we performed H&E staining and IF analysis for the matched tumors and tumor organoids. As shown in Fig. 3, we applied the biomarkers used in the routine pathological diagnosis, including the ductal and basal cell markers (CK5, p63), the glandular and luminal cell markers (CK8, AQP5), and the myoepithelial cell markers (CK5, SMA, p63). Interestingly, these organoids exhibited overlapping growth patterns and cell types, as illustrated in Fig. 3 that the CK5^+^ cells and the CK8^+^ luminal cells comprised the most mass in almost all the organoids. However, the proportion of these two types of cells in tumor mass was different according to the specific subtype of SGTs, which was consistent with the parental tissue (Fig. 4).

In particular, PA, the benign and most frequently occurring tumor in the SGs, consists of glandular cells and myoepithelial cells that are arranged in ducts, sheets, mucous stroma-like, and chondral stroma-like structures [6]. We observed that the PDOs also formed a similar substructure to the parental tumors, and the expression patterns of biomarkers (CK5^+^, CK8^+^, p63^+^, AQP5^+^) were consistent with the parental tumors (Fig. 4 A). BCA is another type of benign tumor in SGs, characterized by basal cell-like tumor cells that are arranged in the typical palisading structures [6]. Interestingly, the organoids exhibited a palisade-like structure with the outer layer of p63^+^ basal cells and the inner layer of CK8^+^ luminal cells together with CK5^+^ ductal cells, which was similar to the original tumors (Fig. 4 B). MEC is the most common malignancy in SGs, featured with squamoid, mucous and intermediate cells with solid and cystic patterns (Fig. 4 C). The squamoid cells (CK5^+^, CK8^+^) and the mucin-producing cells (CK8^+^, AQP5^+^, MUC4^+^) comprised the tumor mass in both PDOs and the parental tumors, and these cells formed typical luminal structure and nest patterns. Like the parental tumors, the outer layer of the solid pattern in PDOs was CK5^+^ p63^+^ basal cells, while the inner layer of tumor mass was CK8^+^ AQP5^+^ luminal cells. The mucus-producing marker MUC4 was positive in both tumor tissue and tumor organoids. ACC is the second most common malignancy in SGs, and the tumor cells are composed of ductal epithelial and myoepithelial cells arranged into tubular, cribriform, and solid structures [5]. Notably, our ACC organoids displayed all these three typical structures which are arranged by luminal cells (CK8^+^, AQP5^+^), basal cells (CK5^+^ p63^+^). (Fig. 4 D). AciCC, the third most common malignancy, comprises of acinar and ductal cells. AciCC organoids showed the small acini structures which were positive for acinar cells markers (CK8^+^, CK5^−^, AQP5^+^), and the known biomarkers (DOG1^+^, SOX10^+^) (Fig. 4 E). These markers in organoids are identical to the parental tumors, indicating that the PDOs maintained the molecular characteristics of AciCC and reconstituted the acinar structure as the original tumors. SDC is another common malignancy in SGs, characterized by comedonecrosis and cribriform in the nest tumor mass. The tumor cells are typically apocrine oncocytoid with abundant cytoplasm and a large pleomorphic nucleus [5]. The cultured organoids showed a solid pattern with the large cytoplasm and nuclei cells, which was consistent with the parental tumors. In both organoids and tumor tissues, CK8^+^ AQP5^+^ cells were the major population, which suggested that the tumor cells in SDC were terminally differentiated ductal cells. Expression pattern of HER2 (a marker for SDC) in the organoids were also consistent with their parental tumors (Fig. 4 F). Compared with SGTs organoids, NSG organoids showed bigger and more regular luminal structures. Similar to the NSG tissue, the inner layer of organoids locates CK8^+^ AQP5^+^ luminal cells, and the outer layer CK5^+^ p63^+^ basal cells (Fig. 4 G). AB-PAS staining showed the secreted mucus in NSG organoids, as well as in AciCC organoids (Fig. 4 G, H).

### PDOs recapitulate the transcriptional features of parental SGTs

**Fig. 5:**
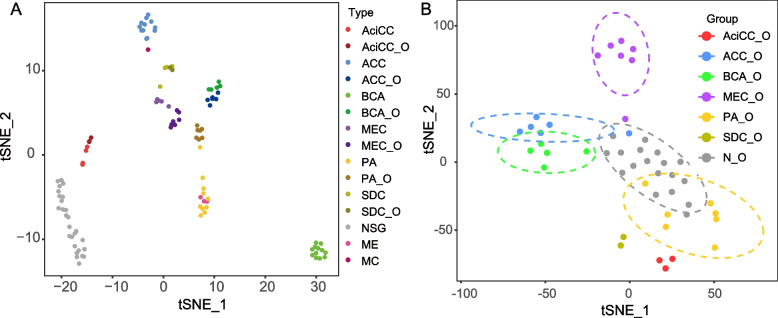
PDOs recapitulate the transcriptional features of parental SGTs. A t-stochastic neighbor embedding (t-SNE) of bulk RNA-seq profiles from NSG (*n* = 27), SGTs (*n* = 51), and the corresponding organoids (*n* = 30). B t-stochastic neighbor embedding (t-SNE) of bulk RNA-seq profiles from the SGTs (n = 30) and NSG (*n* = 18) organoids. The 90% confidence ellipses were applied to show the samples that are more than 4

We performed transcriptome analysis for all these organoids and their corresponding tissue. t-distributed stochastic neighboring embedding (t-SNE) is one of the popular dimensionality reduction techniques to visualize RNA-seq data in two dimensions. By performing t-SNE, the sequencing data of 108 samples were shown in Fig. 5 A, and all the aberrant samples that did not show the subtype-specific characters were excluded in the following analysis. We found that each type of tumor, organoids, and NSG could form its own clusters, and both tumors and their corresponding organoids could be separated from NSG. This suggested that each subtype of SGTs had its own distinct transcriptional characters. Interestingly, the clusters of AciCC, MEC, and SDC were close to their corresponding organoids, indicating that the cultured organoids could maintain the transcript stability as their parental tumors. However, the t-SNE plot revealed the separation of ACC, PA, and BCA from their corresponding organoids. We think the major change in these organoids might be due to the diminishing of myoepithelial cells that are exclusively present in ACC, PA, and BCA. Of note, in the t-SNE plot, we also included 3 cases of myoepithelioma (ME) and one case of myoepithelial carcinoma (MC), whose main cell types are myoepithelial cells to visualize the benign and malignant tumors with myoepithelial cells. Myoepithelial cells have characteristics of both epithelium and smooth muscle cells. It is known that the current method of organoids culture is selective for growing epithelial cells, therefore the myoepithelial cells might gradually diminish under this condition.

To confirm the tumor identity of these SGTs organoids, we performed transcriptome analysis of NSG organoids and SGTs organoids. The t-SNE plot revealed the segregation of normal and tumor organoids into different clusters (Fig. 5 B), which indicates these tumor organoids have their own tumorigenic characteristics, and did not originate from the remaining normal stem cells. We also used specific tumor markers to identify the organoids derived from specific subtypes of SGTs, e.g. MUC4 was used to identify MEC, DOG1 and SOX10 were used to identify AciCC, and HER2 and AR were applied to identify SDC (Fig. 4). However, there is still a lack of specific markers for the other subtypes of SGTs, therefore, we aim to screen the potential biomarkers by using the organoid model in the following work.

### Organoids clustering reveals the epithelial characteristics and visualizes the intrinsic relationship among the subtypes

**Fig. 6:**
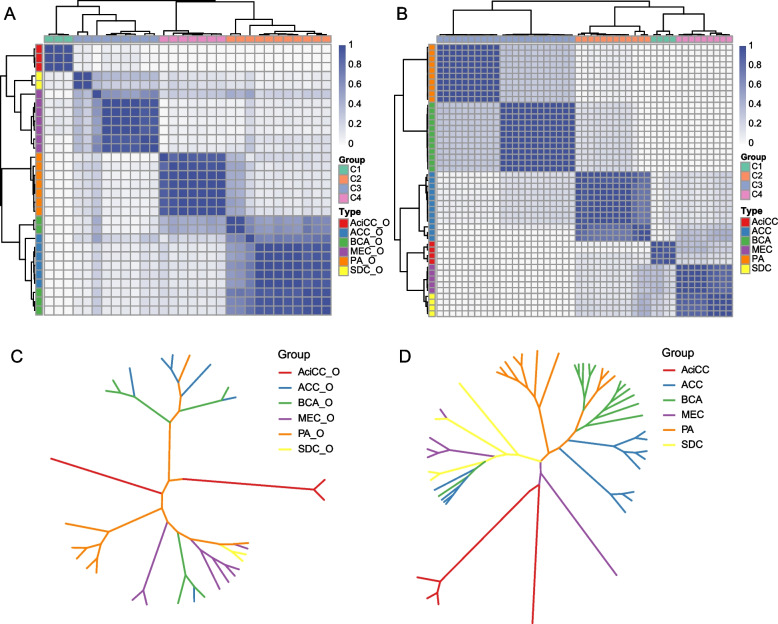
Visualizing the characteristics and the associations among the subtypes based on transcriptomic profiling. A and B Heatmap showed the clustered subgroups of SGTs organoids (n = 30) and SGTs tissue samples (*n* = 48) by subtype consensus clustering analysis. k-means group and consensus clustering groups resulted from the unsupervised algorithm k-means (k = 4) of the 5000 most variable genes. C and D Tree-like structures showed the distribution of 30 SGTs organoids or 48 SGTs tissue samples by hierarchical clustering analysis. Unbiased k-means consensus clustering was performed

To understand the heterogeneity and overlap of SGTs, we need to comprehensively evaluate the molecular characteristics among the subtypes of SGTs. Firstly, we analyze the 6 most common SGTs by performing a K-means clustering with our transcriptomic database. Although these tumors were classified morphologically into 6 subtypes by pathological diagnosis, both the transcriptomic profiling of PDOs and tumor tissue were clearly clustered into 4 subgroups (Fig. 6 A and B). The clusters of organoids model showed a merge of BCA and ACC, but a separation from all other subtypes of SGTs (Fig. 6 A). This suggested a clear overlap of epithelial characteristics between BCA and ACC. Interestingly, with the tumor stroma, the entire tumor tissue model reveals a separation of BCA and ACC (Fig. 6 B), which indicated the stromal characteristics of BCA and ACC are distinct since they could be clustered into different groups. However, MEC and SDC were clustered into the same group in both the organoid and the entire tissue models, but could be slightly separated in the organoid model, which indicated their epithelial characteristics were distinct, but not the stromal characteristics.

To further understand the correlation among these subtypes of SGTs, we applied hierarchical clustering analysis based on Euclidean distance and complete-linkage with the transcriptional profiles, which could visualize the associated data in a tree-like structure [26]. The tree structure in the organoid model showed that PA was associated with all the other subtypes of SGTs, BCA and ACC were closely associated, and MEC and SDC were closely related (Fig. 6 C), which is indeed consistent with the clinical observation. As we know PA, BCA, and ACC are commonly composed of CK5^+^ basal cells, CK8^+^ luminal cells, and SMA^+^ myoepithelial cells, while MEC and SDC are composed of CK5^+^ basal cells and a large number of CK8^+^ luminal cells. Notably, PA is the most plastic subtype of SGTs in the clinic, that frequently undergo malignant transformation, such as MEC, ACC, SDC, or NOS [29]. Of interest, with the input from the tumor stroma, the tree-like structure of the entire tumor tissue showed that BCA, ACC, and PA were related to each other, but BCA and ACC were further separated (Fig. 6 D), which indicates it is the tumor stroma that makes them more different. In addition, MEC and SDC were associated, while AciCC was a distinct branch in the tissue model as well as in the organoid model. This is also consistent with our observation and the other study that AciCC does not directly develop from CK5^+^ stem cells in the organoid model, but might develop through the self-duplication [30].

### Selection of the potential biomarkers in the 6 most common types of SGTs

**Fig. 7:**
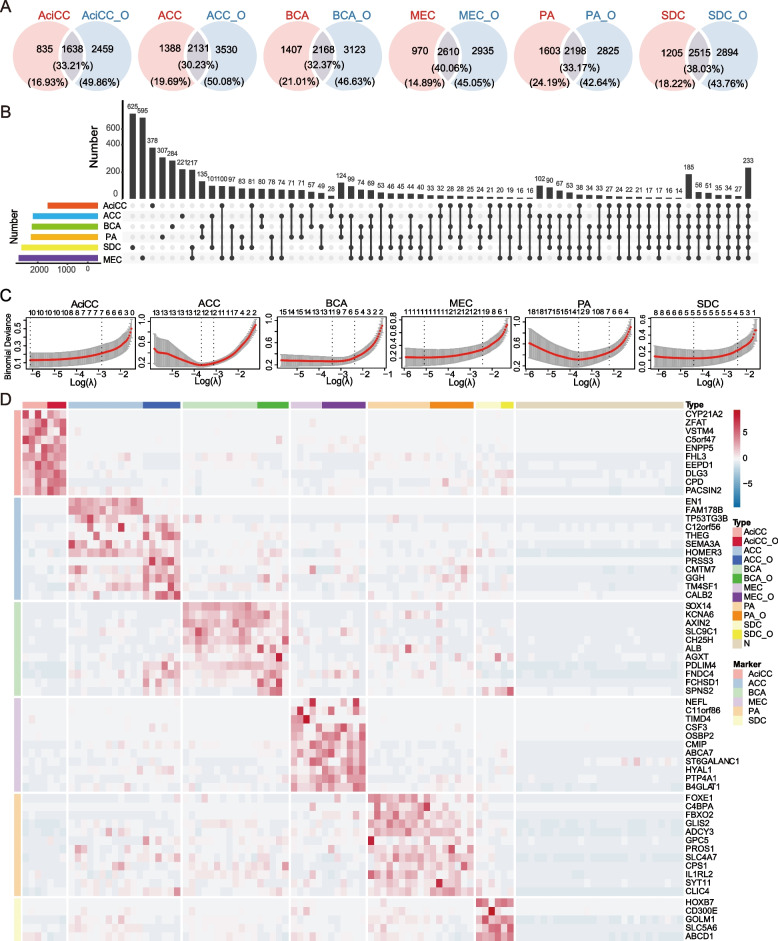
Selection of the potential biomarkers in the 6 most common types of SGTs. A Venn plots showed the characteristic epithelial genes in the differentially expressed genes between tumors and the corresponding organoids (log FC = 1.5, adjusted *P* value < 0.05). B Upset diagram showed the numbers of intersecting genes in the characteristic epithelial genes from six subgroups of SGTs, the left bars showed the number of genes from each tumor. C Lasso analysis of the characteristic genes in six subtypes of SGTs, and the plot showed the number of genes when cross-validation error rates were the lowest. D Heatmap showed the expression of picked characteristic genes from Lasso analysis in SGTs and the corresponding organoids

After understanding the similarities and differences among the subtypes, we aim to select the subtype-specific biomarkers. Since the organoid culture selectively maintains the epithelial cells from the parental tumors, tumor organoids are also an ideal model to identify tumor biomarkers without interference from the tumor stroma. For this purpose, we selected the biomarkers that were exclusively highly expressed in these 6 types of SGTs respectively. Firstly, we picked up the DEGs in each type of tumor tissue and the corresponding organoids by comparing them with the normal tissue. By intersecting analysis, the DEGs presented both in tumor tissue and its organoids were selected, which represented the DEGs mainly in tumor epitheliums (Fig. 7 A). Upset plot (Fig. 7 B) showed that the selected DEGs were exclusively present in the specific type of SGTs. To avoid possible overfitting and multicollinearity in screening the variables in high-dimensional data, we applied the logistic regression model with lasso regularization to define the lowest cross-validation error rates to pick up the most remarkable DEGs (Fig. 7 C). Finally, we selected the potential biomarkers exclusively highly expressed in the 6 most common types of SGTs respectively. These candidates identified in tumor organoids showed similar expression patterns to the parental tumor tissues (Fig. 7 D), which indicated that tumor organoids kept consistent transcriptomic stability in the culture system in vitro. Notably, the level of EN1 and FAM178B were exclusively high in ACC, but not in the corresponding cultured organoids. We speculate these genes might be related to myoepithelial cells, since myoepithelial cells are the main cell components in ACC and diminish in the long-term organoids culture.

### Validation of the potential biomarkers in MEC

**Fig. 8:**
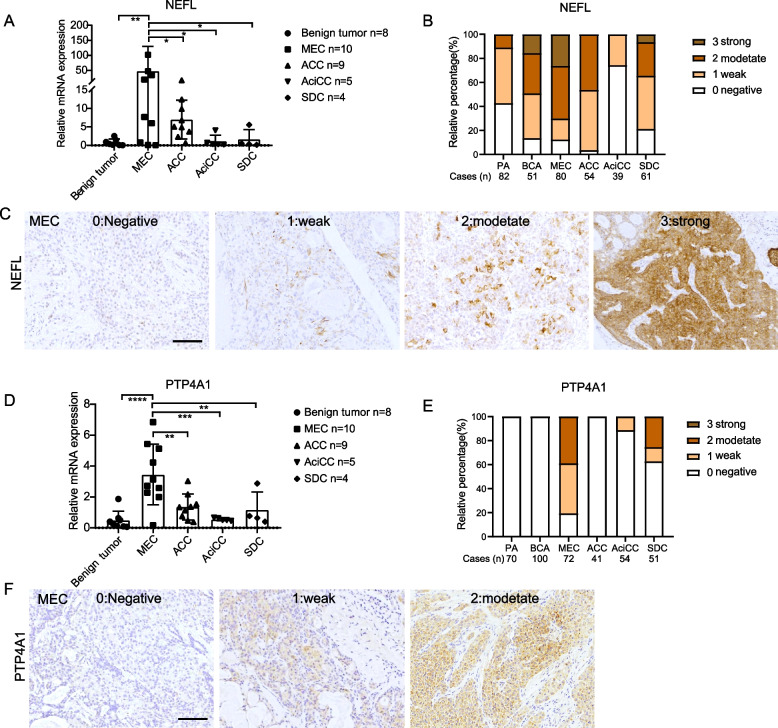
Validation of the potential biomarkers in MEC. A RNA level of NEFL in SGTs by RT-PCR. * *P* < 0.05, ** *P* < 0.01, one-way ANOVA. B Expression of NEFL was detected on TMA sections in a cohort with 367 samples. Immunoreactivity (IR) was scored as negative (IR = 0), weak (IR = 1), moderate (IR = 2), and strong (IR = 3). C The representative images of NEFL in MEC by IHC staining. Scale bar, 100 μm. D RNA level of PTP4A1 in SGTs by RT-PCR. ** *P* < 0.01, *** *P* < 0.001, **** *P* < 0.0001, one-way ANOVA. E Expression of PTP4A1 was detected on TMA sections in a cohort with 388 samples. F The representative images of PTP4A1 in MEC by IHC staining. Scale bar, 100 μm

MEC is the most common malignant tumor in SGTs, characterized by variable cells and structures. It has overlapping morphologic features with cystadenocarcinoma, adenosquamous carcinoma, squamous cell carcinoma, and so on. Many efforts have been made to identify biomarkers to increase diagnostic accuracy [31, 32]. We found here that NEFL and PTP4A1 were the distinct genes highly expressed in MEC compared with other types of SGTs, therefore, both RNA and protein levels of NEFL and PTP4A1 in SGTs were evaluated. By qPCR, NEFL RNA was detected and showed a prominently high level in MEC compared with other types of SGTs (Fig. 8 A), which was concordant with the RNA-seq data. The protein level of NEFL was evaluated by IHC staining on TMA sections, with a panel of 367 cases of SGTs, including PA (*n* = 82), BCA (*n* = 51), ACC (*n* = 54), AciCC (*n* = 39), SDC (*n* = 61), MEC(*n* = 80) respectively. As shown in Fig. 8 B and C, NEFL was highly expressed in both cell membrane and cytoplasm in MEC, and the percentage of immunoreactivity (IR) was strong 26% (21/80), medium positive 44% (35/80), weak staining 18% (14/80), negative 12% (10/80) respectively. However, it could also be detected in PA 11% (9/82), BCA 49% (25/51), ACC 46% (25/54), and DC 35% (21/61) with strong and medium positive IR (Additional file 1 Supplementary Fig. S3A). For PTP4A1, both RNA level and protein level were the highest in MEC (Fig. 8 D, E, and F). IHC staining showed that PTP4A1 was positive in MEC 81% (58/72), including medium positive 39% (28/72), weak staining 42% (30/72), and negative 19% (14/72). Interestingly, there was a lower expression of PTP4A1 in AciCC and DC (Additional file 1 Supplementary Fig. S3B), and all these tumors that expressed PTP4A1 were the non-myoepithelial types of SGTs, which suggested that PTP4A1 might function in tumor epitheliums, but not myoepithelial cells.

We further evaluated both markers in the organoids and their parental tissues by IHC staining. It was shown organoids had similar staining as their parental tissue. NEFL was highly expressed in MEC organoids and their source tissues, while lower expressed in the other subtypes except for AciCC. Remarkably, PTP4A1 functioned as a good potential marker for MEC, which was specifically expressed in MEC and the parental tissues, but not in the other subtypes of SGTs (Additional file 1 Supplementary Fig. 4).

## Discussion

SGTs are a big group of rare tumors, including 11 benign and 23 malignant entities of epithelial origin, and only 2–5% of non-epithelial tumors [33]. The heterogeneous and overlapping features among the subtypes make it difficult for pathological diagnosis. In order to identify clinical diagnostic markers, we need to understand the intrinsic association and the difference among the subtypes. Human organoid models, established from pluripotent stem cells (PSCs) and adult stem cells (ASC), have been widely applied as a robot tool to study tumors [20, 34], since there is a small population of tumor stem cells with self-renewal ability and potential of multi-differentiation [6, 35]. By using the organoids culture technique, we successfully established a series of PDOs of SGTs, including 21 benign tumors and 24 malignant tumors. These PDOs showed similar histomorphological and transcriptomic features to their parental tumors, which brought a chance for us to further study these rare tumors in vitro in a living model. In addition, we tested a method for long-term cryopreservation of PDOs, and the morphological characteristics of organoids could be maintained after a freezing-thawing circle for one and half years (Additional file 1 Supplementary Fig. S5), which enables tumor organoids to recover whenever it needs. These investigations will benefit the establishment of biobanks for rare tumors or other rare diseases.

For culture of SGTs organoids, we followed Driehuis’ protocol which has been widely used for the establishment of tumor organoid library. Lassche et al. recently used the protocol for prostate cancer PDOs to culture SGTs organoids, with additional supplements of R1881 (synthetic androgen) and SB202190 (p38 MAPK inhibitor) [36]. Comparing Lassche’s protocol, we have a higher success rate of organoids culture. More recently, Yoon et.al applied NRG1(neuregulin 1) to take place of EGF in tumor organoid media, to improve the luminal cell components for SGTs organoids. They also optimized the cultural media for NSG organoids, and found retinoic acid could reduce squamous metaplasia, EGF and WNT promote the generation of basal cell-rich organoids, and DAPT improves the differentiation into acinar cells [37]. These findings in NSG organoids, inspired that it is interesting to investigate further if specific signaling trigger specific differentiation in the SGTs organoid model, which could help to answer what determines the subtype-specific phenotype of SGTs.

NSG is characterized by biphasic structures, composed of luminal and abluminal cells **(**Fig. 3). Luminal cells (CK8^+^, AQP5^+^) consist of acinar and ductal cells, while abluminal cells (CK5^+^, SMA^+^, p63^+^) include myoepithelial and basal cells [38]. However, SGTs show multi-generous architectures. Based on the organoids culture, we observed the overlap in growth patterns among the subtypes of SGTs, including solid structures, ductal and glandular patterns, rare cribriform, and scattered tumor cell clusters, which appeared in most of the organoids that were derived from different subtypes of SGTs. This overlapping character in growth patterns actually is common in SGTs, and interestingly, PDOs recapitulated this phenomenon and form similar morphological structures, although they are similar to the parental tumors.

The morphological similarities suggest that the origin of these tumors might come from the same types of stem cells. We observed that most of the SGTs organoids contained plenty of CK5^+^ cells except for AciCC and SDC, while only a few CK8^+^ cells scattered among the CK5^+^ basal cells and formed into the ductal or luminal structures. This might be due to the dual SMAD inhibition applied in the current culture system. Noggin and A83–01 are routinely used to overcome growth arrest and irreversible differentiation, which is efficient to initiate organoids. However, dual SMAD inhibition mainly enables the expansion of epithelial basal stem cells [39]. Practically, Yoon et al. recently formulated a differentiation media, with a lower amount of A83–01, no Noggin, but the addition of DAPT (Notch inhibitor), which could accelerate the differentiation of NSG organoids [37]. Since the current organoid culture is stem cell-derived technology and the tumor organoids are formed mainly through the maintained stem cells, the source of SGTs might originate from those CK5^+^ tumor stem cell-like cells. It is known from the development of NSG that CK5 is a basal stem cell marker, CK5^+^ cells could differentiate into CK8^+^ luminal cells or SMA^+^ myoepithelial cells, important for NSG homeostasis. Centonze et al. reported that CK5^+^ basal stem cells are highly plastic in the differentiation of CK8^+^ luminal cells in physiological conditions, but the communication between luminal cells and basal cells is essential to maintain lineage fidelity in the normal glandular epithelial stem cells [40]. Of interest, we did observe the PDOs established in our study, including NSG and SGTs, mainly consist of three types of cells, CK5^+^CK8^−^ basal cells, CK5^+^CK8^+^ intermediate hybrid cells, and CK5^−^CK8^+^ luminal cells or acinar cells (Fig. 4). We speculate that these CK5^−^CK8^+^ luminal cells are differentiated from CK5^+^ tumor stem cell-like cells. However, whether the CK5^+^ tumor stem cell-like cells originate from the normal CK5^+^ basal stem cells, needs to be further elucidated. It is noteworthy that the current organoid culture medium is only selective for the growth of epithelial cells. The supplemented ROCK inhibitor and TGF-β inhibitor in the media prevent the growth of SMA^+^ myoepithelial cells in the organoid culture (our unpublished data) [41]. As long as the organoids were cultured, fewer and fewer myoepithelial cells could be detected in PDOs, which could be observed clearly in BCA, PA, and ACC that contain myoepithelial cells.

In order to understand the molecular characteristics underlying the heterogenous and overlapping features among the subgroups of SGTs, we performed the subtype consensus clustering and hierarchical clustering analysis. Our data show an overlap of transcriptomic profiles between BCA and ACC in the organoid model, but clear segregation in the entire tissue model. Opposite results were seen for MEC and SDC. These data suggest an overlap apparently in epithelial characteristics between BCA and ACC, while overlap in stroma characteristics between MEC and SDC. We also applied a tree-structured model to visualize the characteristics and the association among the subtypes of SGTs (Fig. 6 C and D). Interestingly, compared with the bulk tissue model, the organoid model remarkably reveals the correlation among the subtypes and shows that PA is the most plastic subtype correlated to all the other subtypes. The organoid model originates from the stem cells and imitates the early stage of tumorigenesis, so we speculate PA might be a transitional stage for those SGTs that undergo a malignant transformation. These malignant subtypes gradually take place of the original PA, consequently shown as initial malignancy in clinic (Fig. 6 C**)**. This is the first time to get direct evidence by bioinformatic analysis, since we generally know there have overlap features among the subtypes of SGTs, and PA frequently undergoes malignancy transformation, such as MEC, ACC, SDC, and NOS [29].

Because the overlap among the subtypes causes difficulty in pathological diagnosis, we sought to find exclusive biomarkers for different subtypes. By comparative analysis, a group of potential biomarkers was identified. Here, we focus on NEFL and PTP4A1 in MEC, since MEC is the most common malignancy but is less characterized. Neurofilament light chain (NEFL) was implicated in the gastric cancer [42] and breast cancer [43]. In this study, we found that NEFL RNA is remarkably high in MEC, corresponding to the high level of protein level in MEC detected by IHC staining. However, NEFL is also detected in PA, ACC, BCA, and SDC, which could limit its application as a diagnostic marker. Protein tyrosine phosphatase 4A1 (PTP4A1), another potential marker in MEC, is found overexpressed in cervical cancer, prostate cancer, pancreatic cancer, colorectal cancer, gastric cancer, liver cancer, and esophageal cancer [44]. We found that both RNA and protein levels of PTP4A1 are higher in MEC compared with the other subtypes of SGTs. As PTP4A1 is specifically expressed in MEC, it could be used to assist pathological diagnosis.

The other potential biomarkers are also promising, such as EN1 and FAM178B in ACC, ZFAT in AciCC, and HOXB7 in SDC. Homeobox protein engrailed-1(EN1), a homeodomain-containing transcription factor, plays a vital role in embryonic development and tumor progress [45–47]. We found that EN1 is exclusively highly expressed in ACC, which is consistent with Baba et al. report that EN1 is a promising diagnostic marker for ACC [48]. Family with sequence similarity 178 member B (FAM178B), highly expressed in ACC and was identified as a potential biomarker (Fig. 7 D). It is a protein-coding gene with an unrecorded function, and a recent transcriptome-wide study identified FAM178B as a novel susceptibility gene for glioma [49]. However, the role of FAM178B in ACC is unknown, and it is interesting to further investigate its potential as a clinical diagnostic marker for ACC. In AciCC, the highest expressed genes included zinc finger and AT-hook domain containing (ZFAT), reported as a crucial transcription factor in primitive hematopoiesis and angiogenesis. HOXB7, a homeodomain (HOX) transcription factor that is important for regional body patterning of invertebrates and vertebrates, is exclusively highly expressed in SDC. HOXB7 was reported as a hallmark in the glioma [50], lung cancer [51], and breast cancer [52], and its role in SDC needs to be further elucidated.

## Conclusions

In this study, we established the first library of PDOs with multiple subtypes of SGTs, and demonstrates PDOs recapitulate the morphological and transcriptional characters of the parental tumors. With the organoid model, cluster analysis brings a chance for us to understand the intrinsic associations and the substantial differences among the subtypes of SGTs. The tree-like structure by the hierarchical clustering analysis accurately reflects the characteristics of SGTs in clinic. This living biobank offers a valuable platform to uncover the potential biomarkers, and further explore and understand the characteristics of these rare tumors.

## Supplementary Information

**Additional file 1.** Fig. S1. The cases and the subtypes of SGTs that failed to culture organoids. A H&E staining of the cases that failed to set up organoids. Scale bars, 100 μm. B and C Warthin tumor and oncocytoma failed to form organoids. Scale bars, 100 μm. Fig. S2. Growth kinetics of SGTs organoids. The representative image of SGTs organoids with brightfield microscope at indicated time points. The enlarged images in boxes showed the detailed structures. Scale bars, 100 μm. Fig. S3. Expression of NEFL and PTP4A1 in SGTs. A and B The representative image of NEFL and PTP4A1 expressed in PA, BCA, ACC, AciCC, SDC by IHC staining. Immunoreactivity (IR) was scored as negative (IR = 0), weak (IR = 1), moderate (IR = 2), and strong (IR = 3). Scale bar, 100 μm. Fig. S4. IHC staining of NEFL and PTP4A1 in SGTs organoids as compared to its parental tissues. Scale bar, 50 μm. Fig. S5. Images of ACC organoids before frozen and after thawed. Scale bar, 100 μm.

## Data Availability

The datasets supporting the conclusions of this article are included within the article and its additional files.

## Abbreviations

SGTs: Salivary gland tumors
PDOs: Patient-derived organoids
NSG: Normal salivary gland
SGs: Salivary glands
PA: Pleomorphic adenoma
BCA: Basal cell adenoma
ACC: Adenoid cystic carcinoma
MEC: Mucoepidermoid carcinoma
AciCC: Acinic cell carcinoma
SDC: Salivary duct carcinoma
ME: Myoepithelioma
MC: Myoepithelial carcinoma
SGC: Submandibular gland cyst
F: Female
M: Male
PG: Parotid gland
SMG: Submandibular gland
SLG: Sublingual gland
MSG: Minor salivary glands
IF: Immunofluorescence
IHC: Immunohistochemistry
FGF: Fibroblast growth factor
EGF: Epidermal Growth Factor
FBS: Fetal bovine serum
CK5: Cytokeratin 5
CK8: Cytokeratin 8
AQP5: Aquaporin 5
SMA: Alpha-smooth muscle actin
HER-2: C-erbB-2
AR: Androgen receptor
TMA: Tissue microarray
tSNE: t-stochastic neighboring embedding
DEGs: Differentially expressed genes
PTP4A1: Protein tyrosine phosphatase 4A1
NEFL: Neurofilament light chain
EN1: Homeobox protein engrailed-1
FAM178B: Family with sequence similarity 178 member B
ZFAT: Zinc finger and AT-hook domain containing
HOXB7: Homeobox B7.
