# Supplementary material for: An organoid library of salivary gland tumors reveals subtype-specific characteristics and biomarkers
Source: J Exp Clin Cancer Res. 2022 Dec 17;41:350. doi: 10.1186/s13046-022-02561-5 (PMC9758872; doi:10.1186/s13046-022-02561-5)
Supplement: Supplementary file 1 — Additional file 1. Fig. S1. The cases and the subtypes of SGTs that failed to culture organoids. A H&E staining of the cases that failed to set up organoids. Scale bars, 100 μm. B and C Warthin tumor and oncocytoma failed to form organoids. Scale bars, 100 μm. Fig. S2. Growth kinetics of SGTs organoids. The representative image of SGTs organoids with brightfield microscope at indicated time points. The enlarged images in boxes showed the detailed structures. Scale bars, 100 μm. Fig. S3. Expression of NEFL and PTP4A1 in SGTs. A and B The representative image of NEFL and PTP4A1 expressed in PA, BCA, ACC, AciCC, SDC by IHC staining. Immunoreactivity (IR) was scored as negative (IR = 0), weak (IR = 1), moderate (IR = 2), and strong (IR = 3). Scale bar, 100 μm. Fig. S4. IHC staining of NEFL and PTP4A1 in SGTs organoids as compared to its parental tissues. Scale bar, 50 μm. Fig. S5. Images of ACC organoids before frozen and after thawed. Scale bar, 100 μm. [file 13046_2022_2561_MOESM1_ESM.pdf]

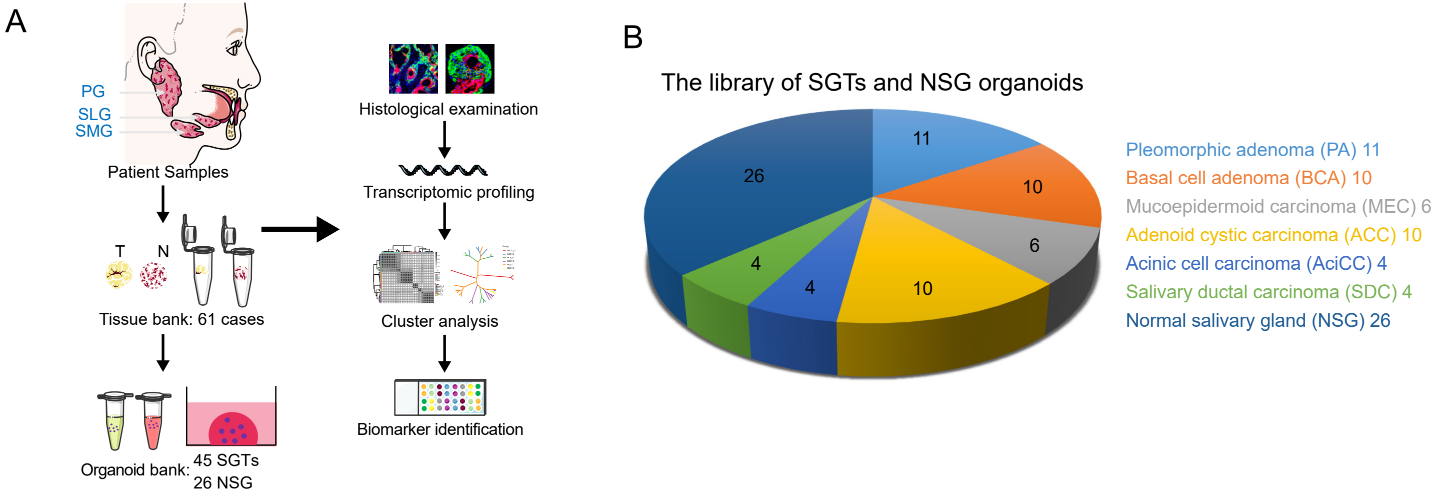

Fig. 1

Establishment of PDOs library of SGTs. A A workflow for this study. **B** Pie chart showed the subtypes of SGTs and NSG organoids in the library.

Table 1. The list of the patient biobank

ID	Sex	Age	Tumor source	TNM	Type	Tissue Normal	Tissue Tumor	3D Organoids Normal	/Passage	3D Organoids Tumor	/Passage	Subtype/ Differentiation
1	M	41	SMG		PA	+	+	+	4	+	5	
2	F	32	SMG		PA	+	+	+	3	+	4	
3	M	55	SMG		PA	+	+	+	5	+	4	
4	M	63	PG		PA		+					
5	F	31	SMG		PA	+		+	4	+	5	
6	M	40	PG		PA	+	+	+	5	+	3	
7	F	43	PG		PA		+					
8	F	57	SMG		PA	+	+	+	5	+	5	
9	F	43	PG		PA	+	+	+	4	+	6	
10	F	64	SMG		PA	+	+	+	4	+	5	
11	F	40	PG		PA	+	+	+	5	+	5	
12	M	20	SMG		PA	+	+	+	5	+	4	
13	M	83	PG		PA	+		+	5	+	5	
14	F	44	PG		BCA	+	+			+	6	
15	F	66	PG		BCA	+	+	+	5	+	5	
16	M	74	PG		BCA	+	+	+	4	+	6	
17	M	56	PG		BCA					+	5	
18	F	64	PG		BCA	+	+	+	3	+	4	
19	F	61	PG		BCA	+	+			+	5	
20	F	59	PG		BCA	+	+	+	5	+	5	
21	F	47	PG		BCA	+	+	+	2	+	5	
22	F	56	PG		BCA	+		+	5	+	5	
23	F	59	PG		BCA	+	+			+	4	
24	M	23	PG	T2N0M0	MEC	+	+			+	5	Moderate
25	F	35	PG	T2N0M0	MEC		+					Moderate
26	F	53	MSG	T1N0M0	MEC		+					Moderate
27	M	33	PG	T1N0M0	MEC		+			+	4	Moderate
28	M	62	PG	T2N0M0	MEC	+	+			+	4	Well
29	M	46	PG	T2N0M0	MEC		+			+	3	Well
30	M	31	PG	T2N0M0	MEC	+	+	+	3	+	5	Well
31	F	62	SMG	T1N0M0	MEC	+	+			+	5	Moderate
32	F	79	SMG	T2N0M0	ACC		+			+	5	Tubular cribriform
33	F	54	SMG	T2N0M0	ACC	+	+			+	5	Cribriform tubuular
34	M	66	MSG	T1N0M0	ACC		+			+	3	Cribriform
35	F	43	SMG	T1N0M0	ACC		+			+	6	Tubular solid cribriform
36	F	60	SMG	T1N0M0	ACC		+					Cribriform tubular
37	M	48	MSG	T1N0M0	ACC		+			+	5	Tubular
38	F	60	SLG	T2N0M0	ACC		+					Tubular,cribriform
39	F	57	SMG	T1N0M0	ACC		+			+	5	cribriforf tubuular
40	F	47	MSG	T2N0M0	ACC	+	+	+	4	+	4	Cribriform
41	F	56	MSG	T2N0M0	ACC		+			+	6	Tubular cribriform
42	M	55	MSG	T2N0M0	ACC		+			+	5	Tubular cribriform
43	F	35	MSG	T2N0M0	ACC					+	5	Tubular cribriform
44	F	34	PG	T2N0M0	AciCC	+	+	+	5	+	3	Acinar
45	F	53	PG	T2N0M0	AciCC	+	+			+	2	Acinar
46	F	25	PG	T2N0M0	AciCC		+	+	5	+	3	Acinar
47	M	29	PG	T2N0M0	AciCC		+			+	2	Acinar
48	M	58	PG	T2N0M0	SDC	+	+	+	3			
49	M	65	SMG	T4N0M0	SDC	+	+	+	5	+	5	
50	F	43	SMG	T4N0M0	SDC	+	+	+	4	+	4	
51	M	53	PG	T4N0M0	SDC	+	+	+	5	+	5	
52	M	39	PG	T2N0M0	SDC	+	+	+	4	+	5	
53	M	47	PG		ME		+					
54	F	65	MSG		ME		+					
55	M	10	MSG		ME		+					
56	M	58	PG	T2N0M0	MC	+	+					Clear cell type
57	F	16	SLG		SGC	+						
58	F	48	SLG		SGC	+						
59	F	10	SLG		SGC	+						
60	F	32	SLG		SGC	+						
61	M	48	SLG		SGC	+						

Abbreviations: F, female; M, male; PG, parotid gland; SMG, submandibular gland; SLG, sublingual gland; MSG, minor salivary glands; PA, pleomorphic adenoma; BCA,basal cell adenoma; MEC,mucoepidermoid carcinoma; ACC,adenoid cystic carcinoma; Acicc, acinic cell carcinoma; SDC, salivary ductal carcinoma; ME, myoepithelioma; MC, myoepithelial carcinoma; SGC, submandibular gland cyst.
+,successfully collected.

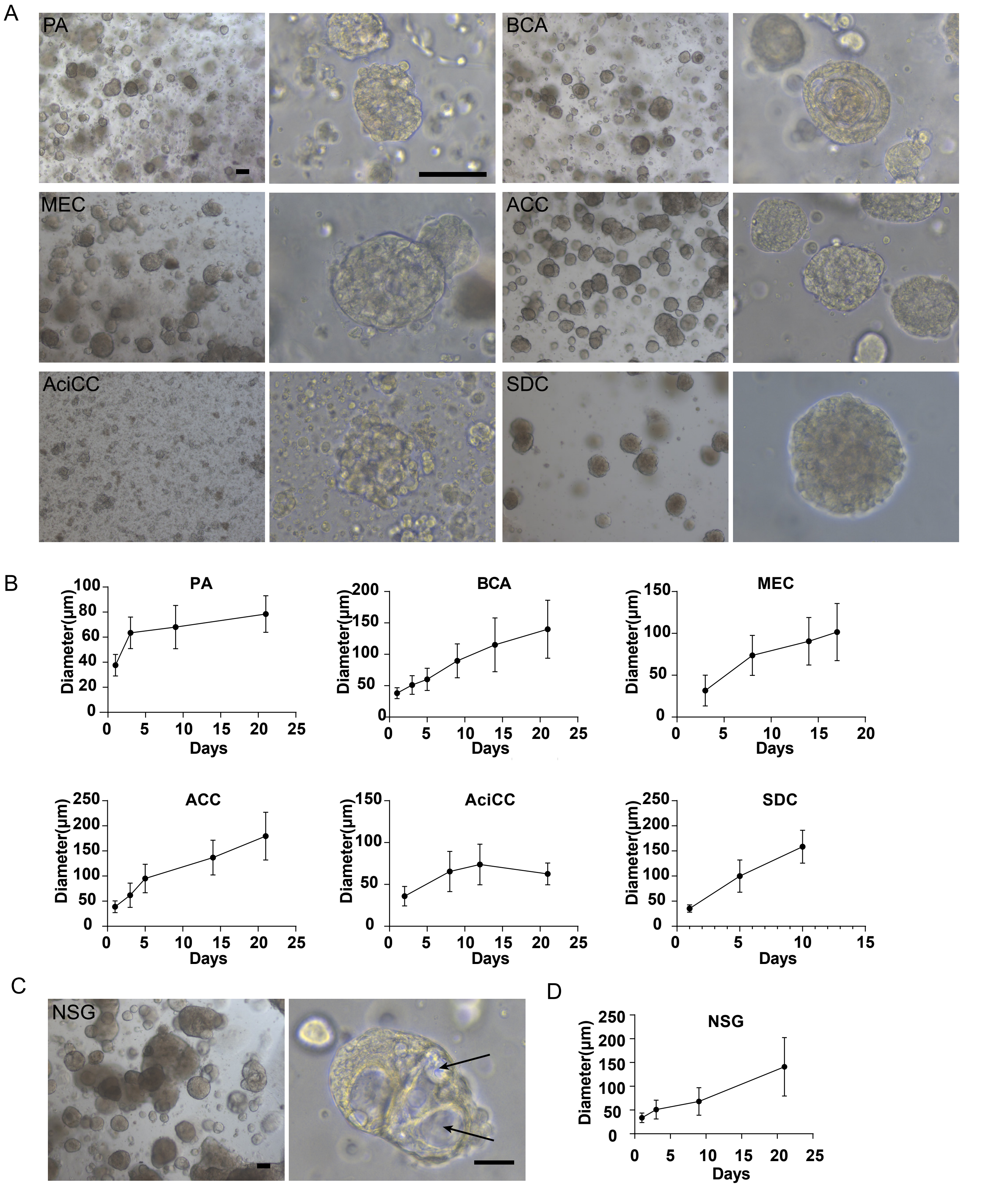

Fig. 2

Structure and growth characteristics of PDOs. A Representative bright-field images of SGTs organoids. Scale bar, 100 μm. **B** Growth kinetics were analyzed by quantifying the average size (μm, mean ± S.D.) of 50 organoids from three independent samples. **C** Representative bright-field images of NSG, and the arrow pointed to the mucus secreted by NSG organoids. Scale bar, 100 μm. **D** Growth kinetics of NSG organoids from three independent samples.

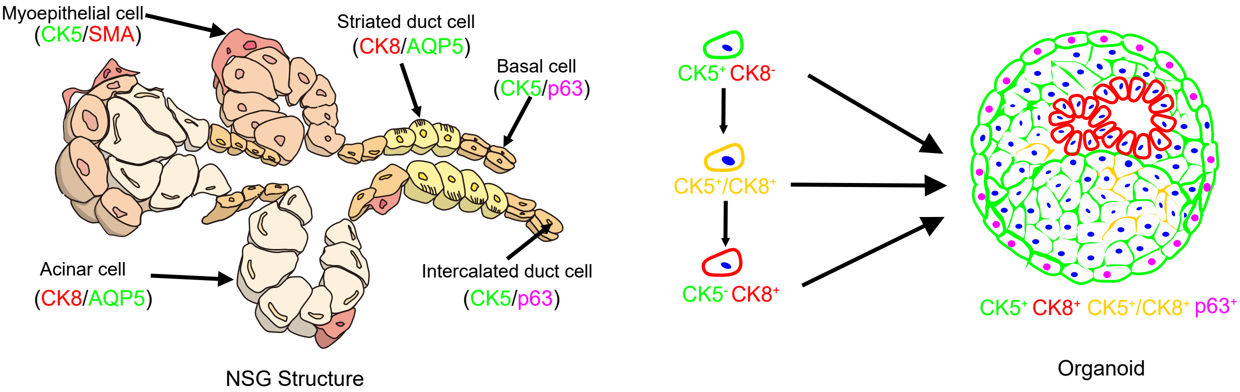

Fig. 3
Schematic diagram showed the cell types and the biomarkers observed in this study.

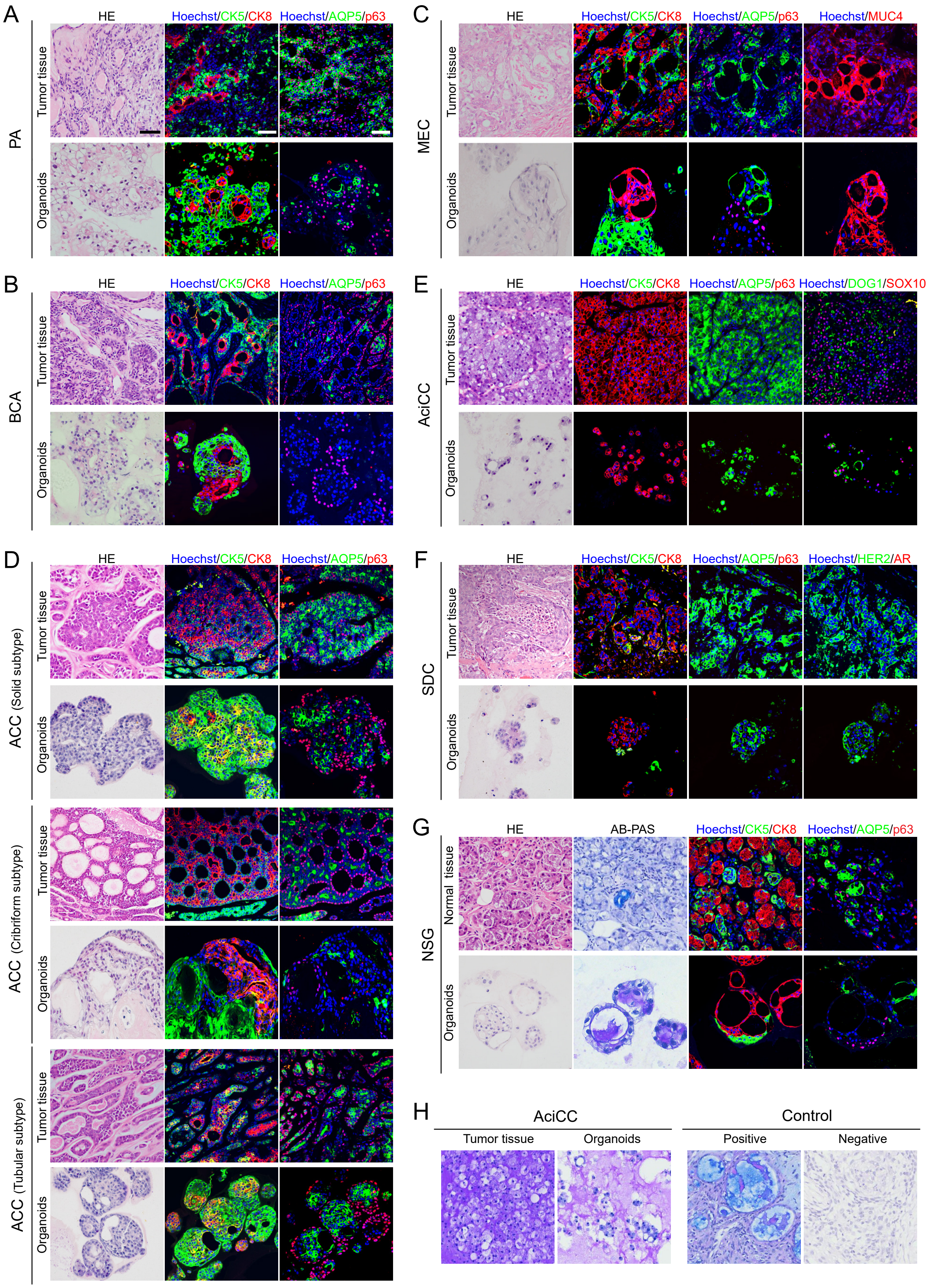

Fig. 4

PDOs recapitulate the morphologic features of parental SGTs. Representative H&E images and confocal images of IF staining with the original tissues and the corresponding organoids. Two benign tumors PA **(A)**, BCA **(B)**; four malignant tumors MEC **(C)**, ACC **(D)**, AciCC **(E)**, SDC **(F)** and NSG **(G);** AB-PAS staining of secreted mucus in the NSG **(G)** and AciCC **(H)**.Clinical diagnostic biomarkers were used to evaluate the morphological and molecular features, including Cytokeratin 5 (CK5),Cytokeratin 8 (CK8), Aquaporin 5 (AQP5), p63, C-erbB-2 (HER2), Androgen Receptor (AR), MUC4, DOG1, SOX10. Scale bar, 50 μm.

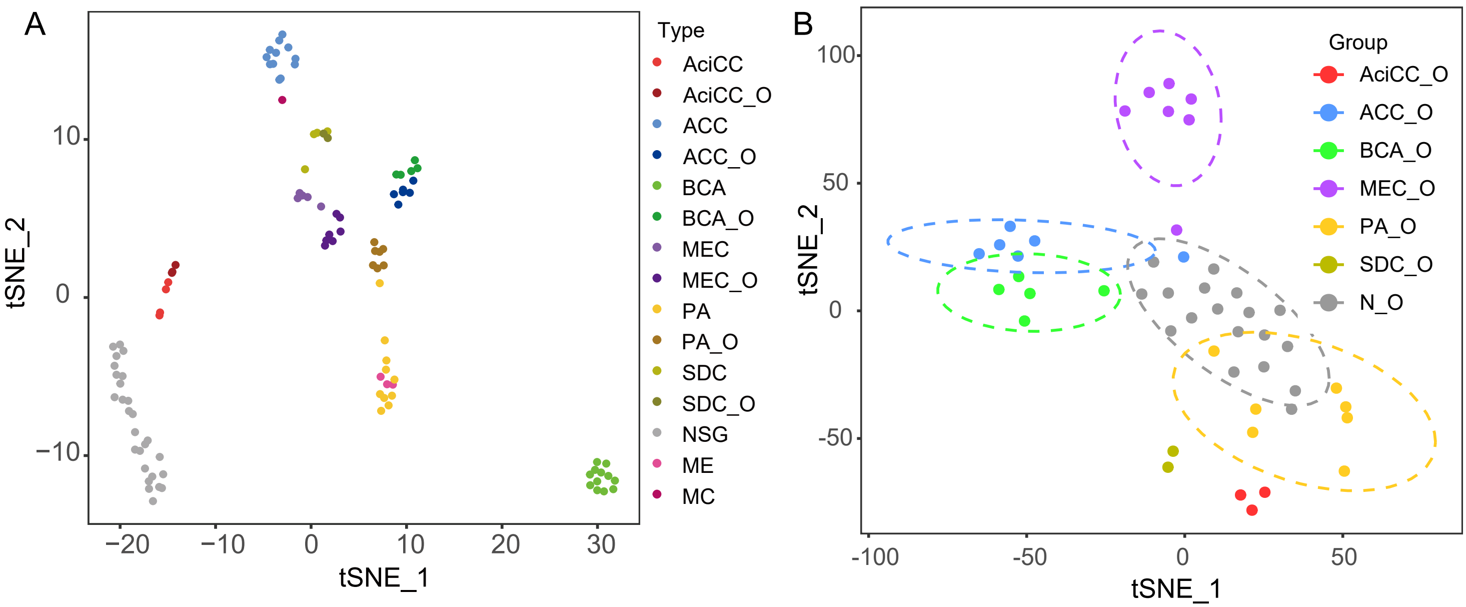

Fig. 5

PDOs recapitulate the transcriptional features of parental SGTs. A t-stochastic neighbor embedding (t-SNE) of bulk RNA-seq profiles from NSG (n=27), SGTs (n=51), and the corresponding organoids (n=30). **B** t-stochastic neighbor embedding (t-SNE) of bulk RNA-seq profiles from the SGTs (n=30) and NSG (n=18) organoids.The 90% confidence ellipses were applied to show the samples that are more than 4.

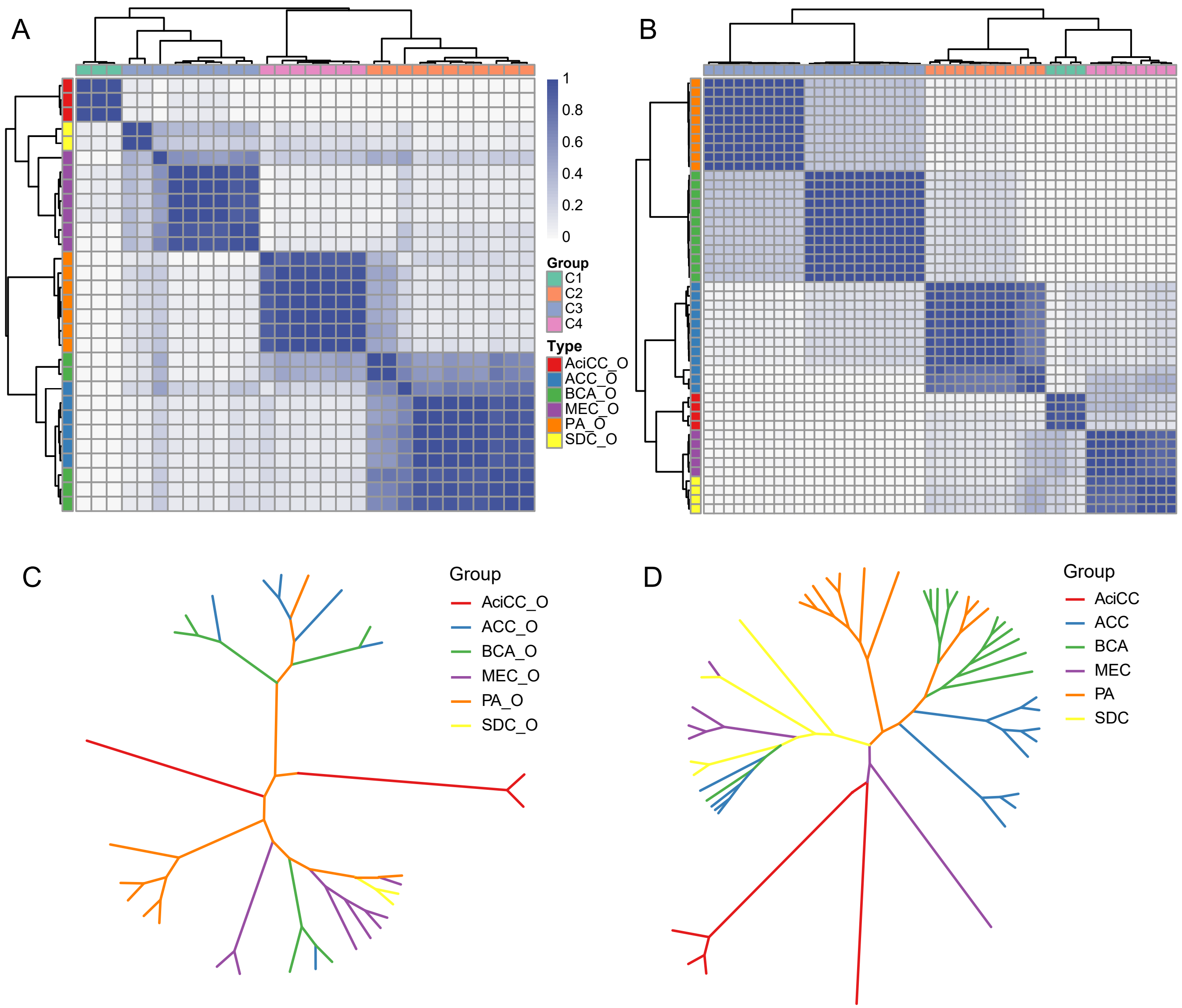

Fig. 6

Visualizing the characteristics and the associations among the subtypes based on transcriptomic profiling. A and **B** Heatmap showed the clustered subgroups of SGTs organoids (n=30) and SGTs tissue samples (n=48) by subtype consensus clustering analysis. k-means group and consensus clustering groups resulted from the unsupervised algorithm k-means (k=4) of the 5000 most variable genes. **C** and **D** Tree-like structures showed the distribution of 30 SGTs organoids or 48 SGTs tissue samples by hierarchical clustering analysis. Unbiased k-means consensus clustering was performed.

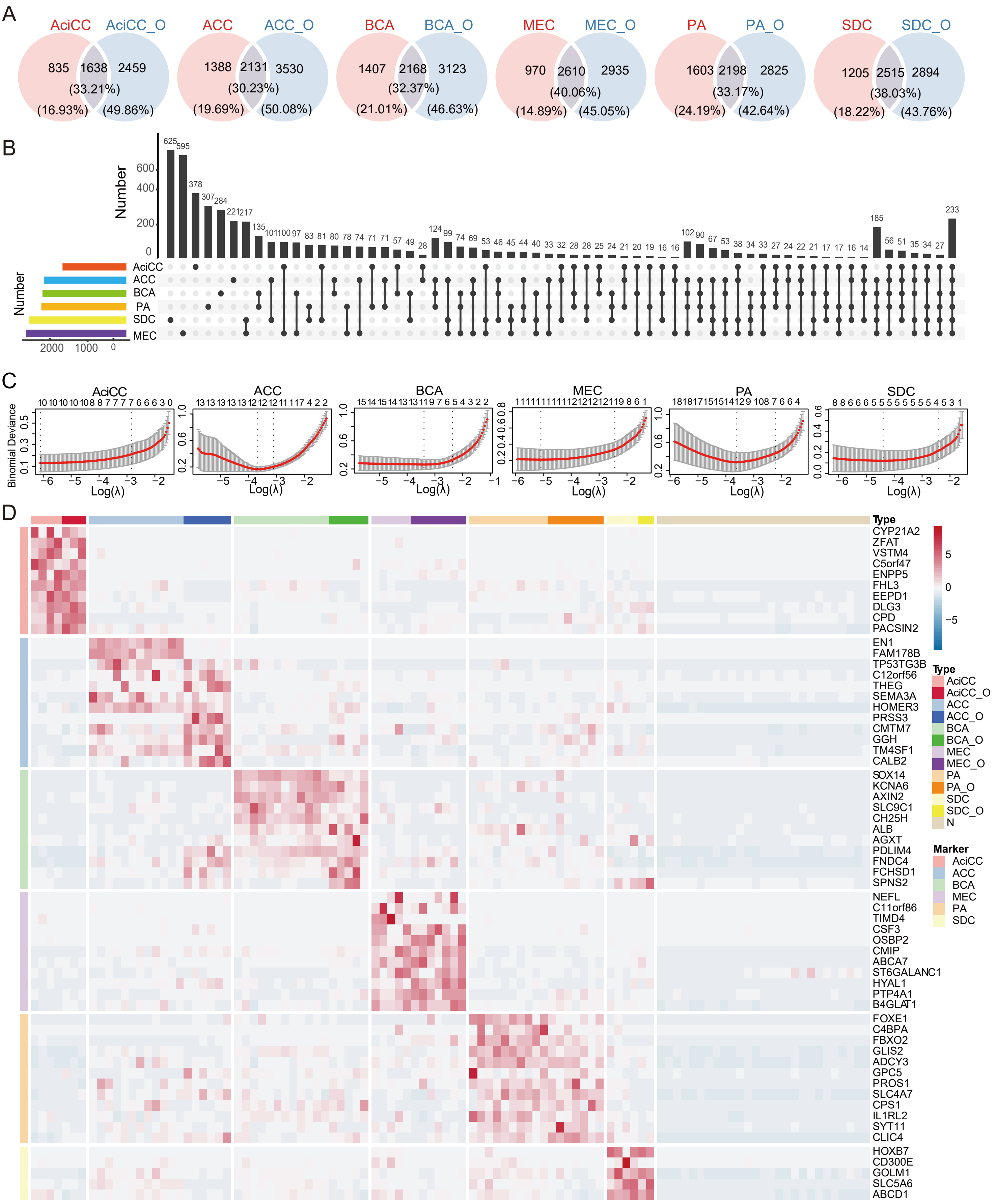

Fig. 7

Selection of the potential biomarkers in the 6 most common types of SGTs. A Venn plots showed the characteristic epithelial genes in the differentially expressed genes between tumors and the corresponding organoids (log FC=1.5, adjusted P value <0.05). **B** Upset diagram showed the numbers of intersecting genes in the characteristic epithelial genes from six subgroups of SGTs, the left bars showed the number of genes from each tumor. **C** Lasso analysis of the characteristic genes in six subtypes of SGTs, and the plot showed the number of genes when cross-validation error rates were the lowest. **D** Heatmap showed the expression of picked characteristic genes from Lasso analysis in SGTs and the corresponding organoids.

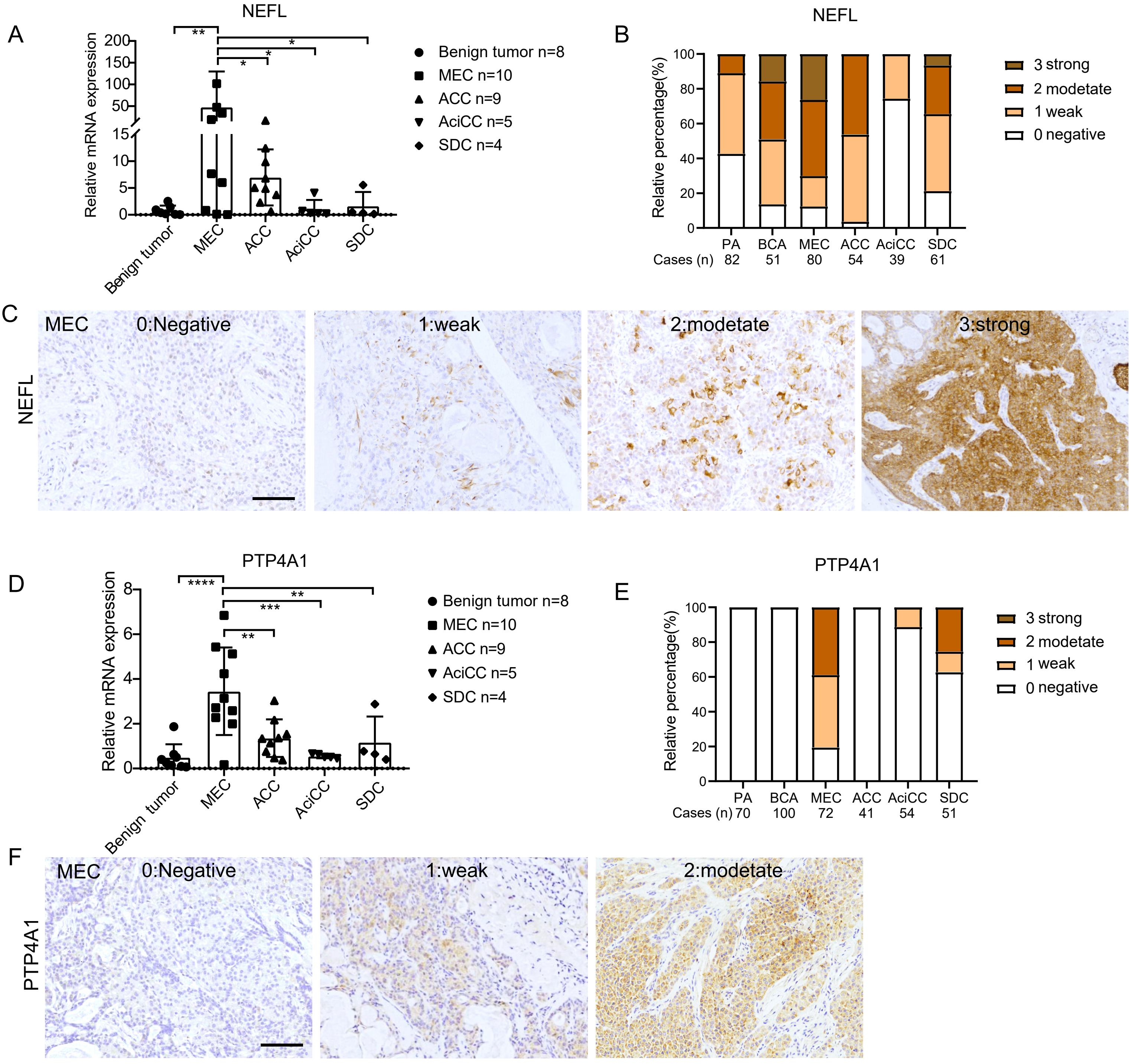

Fig. 8

Validation of the potential biomarkers in MEC. A RNA level of NEFL in SGTs by qPCR. * P <0.05, ** P <0.01, one-way ANOVA. **B** Expression of NEFL was detected on TMA sections in a cohort with 367 samples.Immunoreactivity (IR) was scored as negative (IR = 0), weak (IR = 1), moderate (IR = 2), and strong (IR = 3). **C** The representative images of NEFL in MEC by IHC staining. Scale bar, 100 μm. **D** RNA level of PTP4A1 in SGTs by qPCR. ** P <0.01, *** P < 0.001, **** P < 0.0001, one-way ANOVA. **E** Expression of PTP4A1 was detected on TMA sections in a cohort with 388 samples. **F** The representative images of PTP4A1 in MEC by IHC staining. Scale bar, 100 μm.

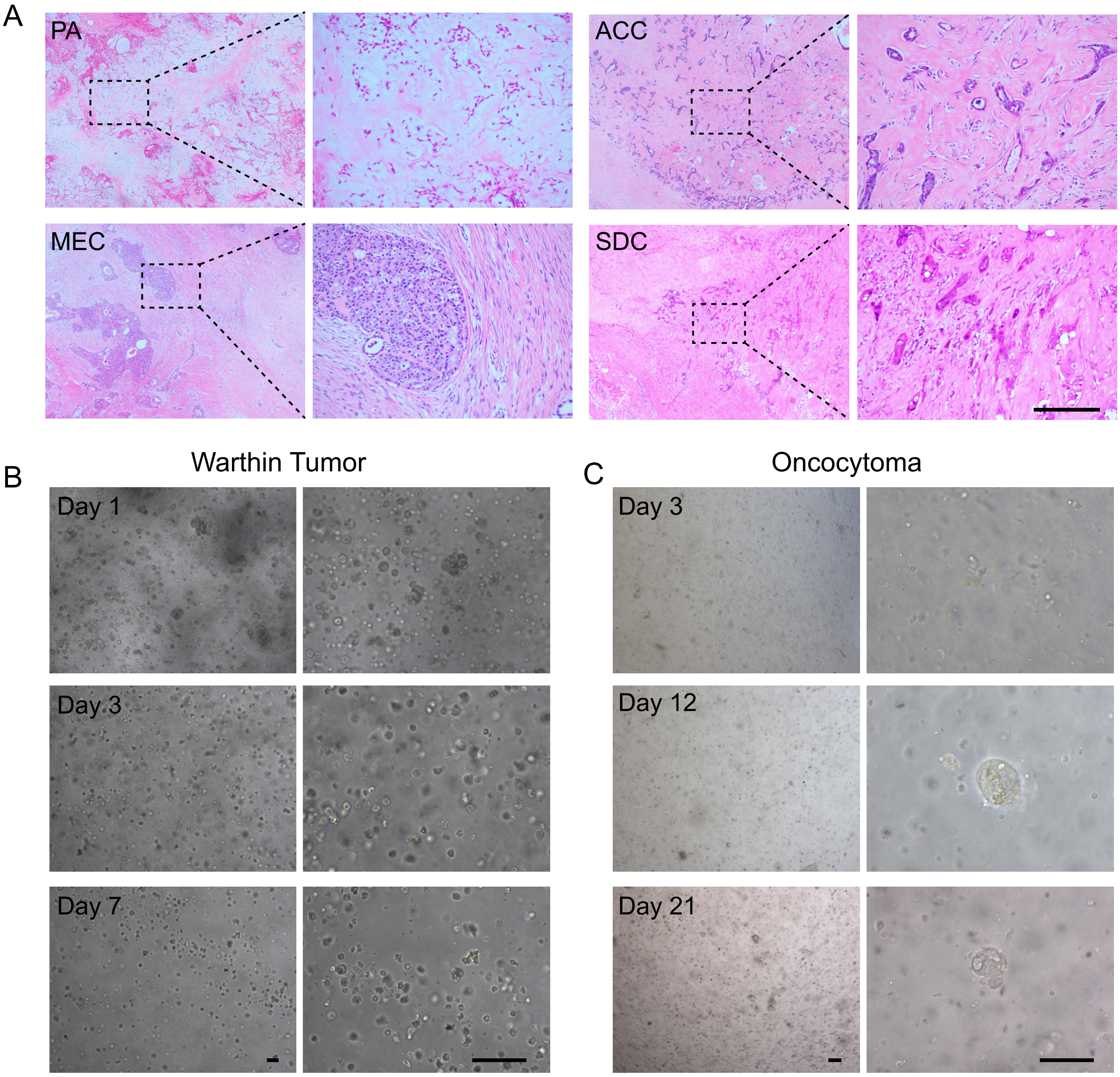

Supplementary Figure S1.
The cases and the subtypes of SGTs that failed to culture organoids. A H&E staining of the cases that failed to set up organoids. Scale bars, 100 μm. **B and C** Warthin tumor and oncocytoma failed to form organoids. Scale bars, 100 μm.

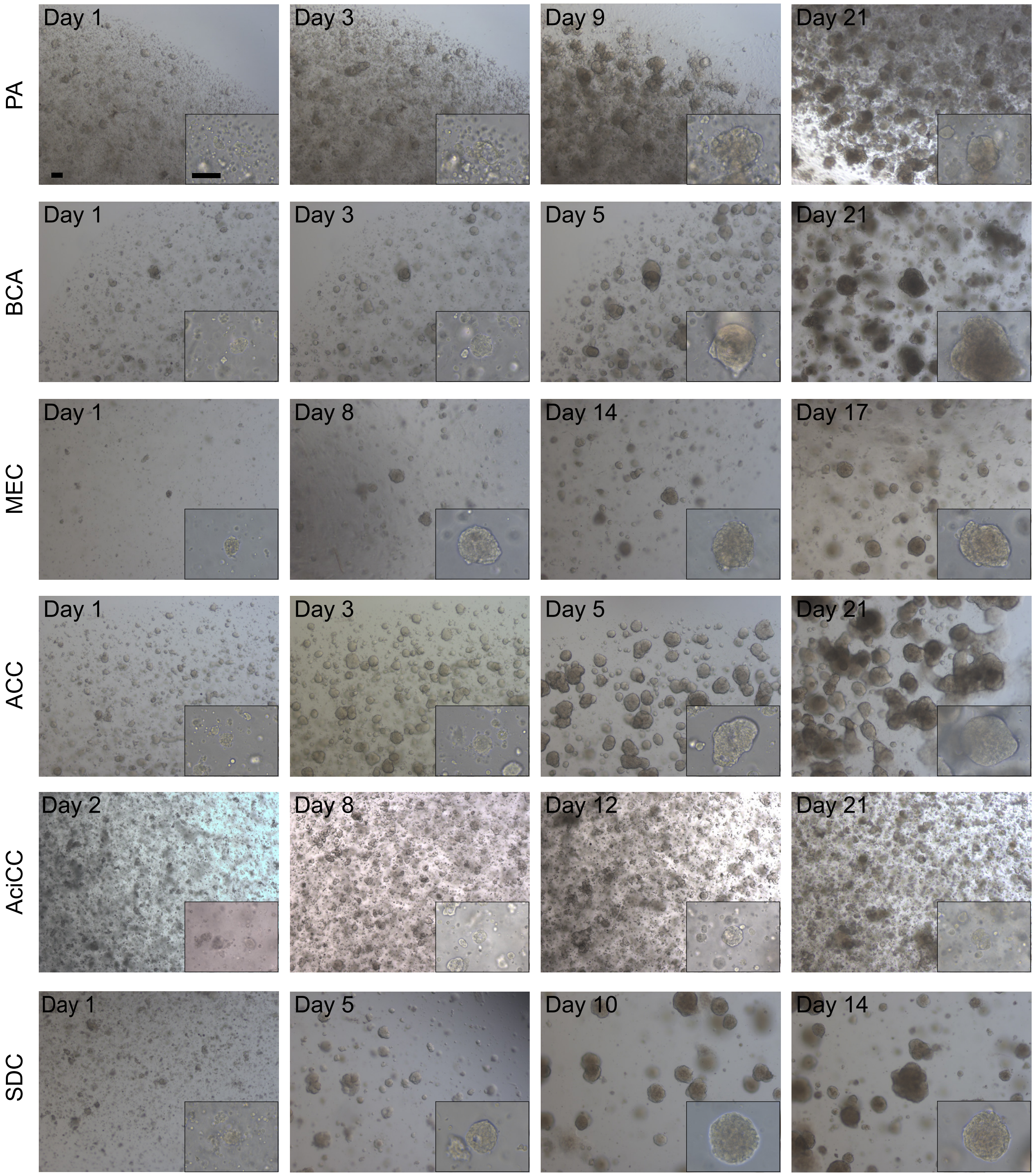

Supplementary Figure S2.

Growth kinetics of SGTs organoids. The representative image of SGTs organoids with brightfield microscope at indicated time points. The enlarged images in boxes showed the detailed structures. Scale bars, 100 μm.

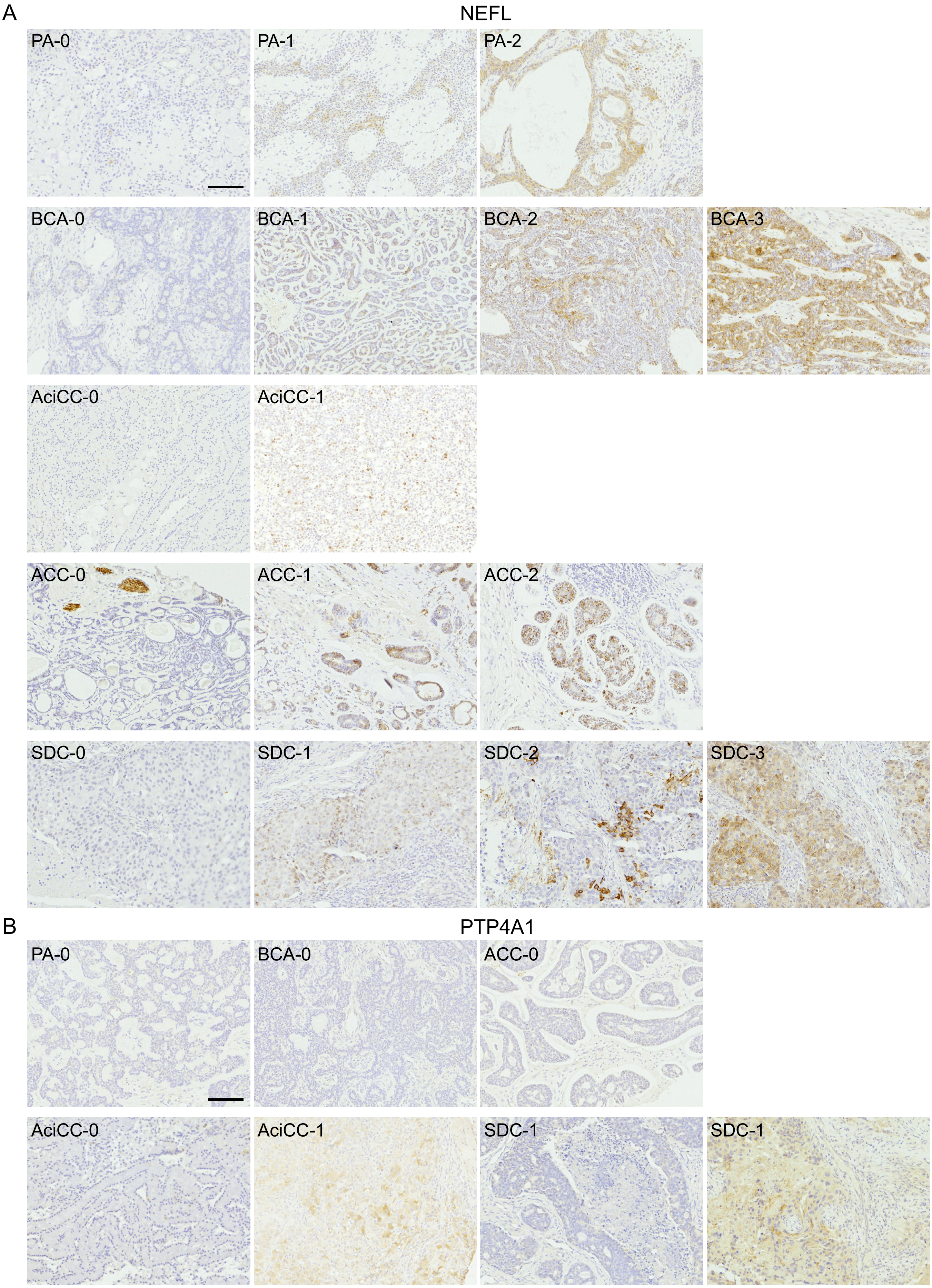

Supplementary Figure S3.

Expression of NEFL and PTP4A1 in SGTs. A and B The representative image of NEFL and PTP4A1 expressed in PA, BCA, ACC, AciCC, SDC by IHC staining. Immunoreactivity (IR) was scored as negative (IR = 0), weak (IR = 1), moderate (IR = 2), and strong (IR = 3). Scale bar, 100 μm.

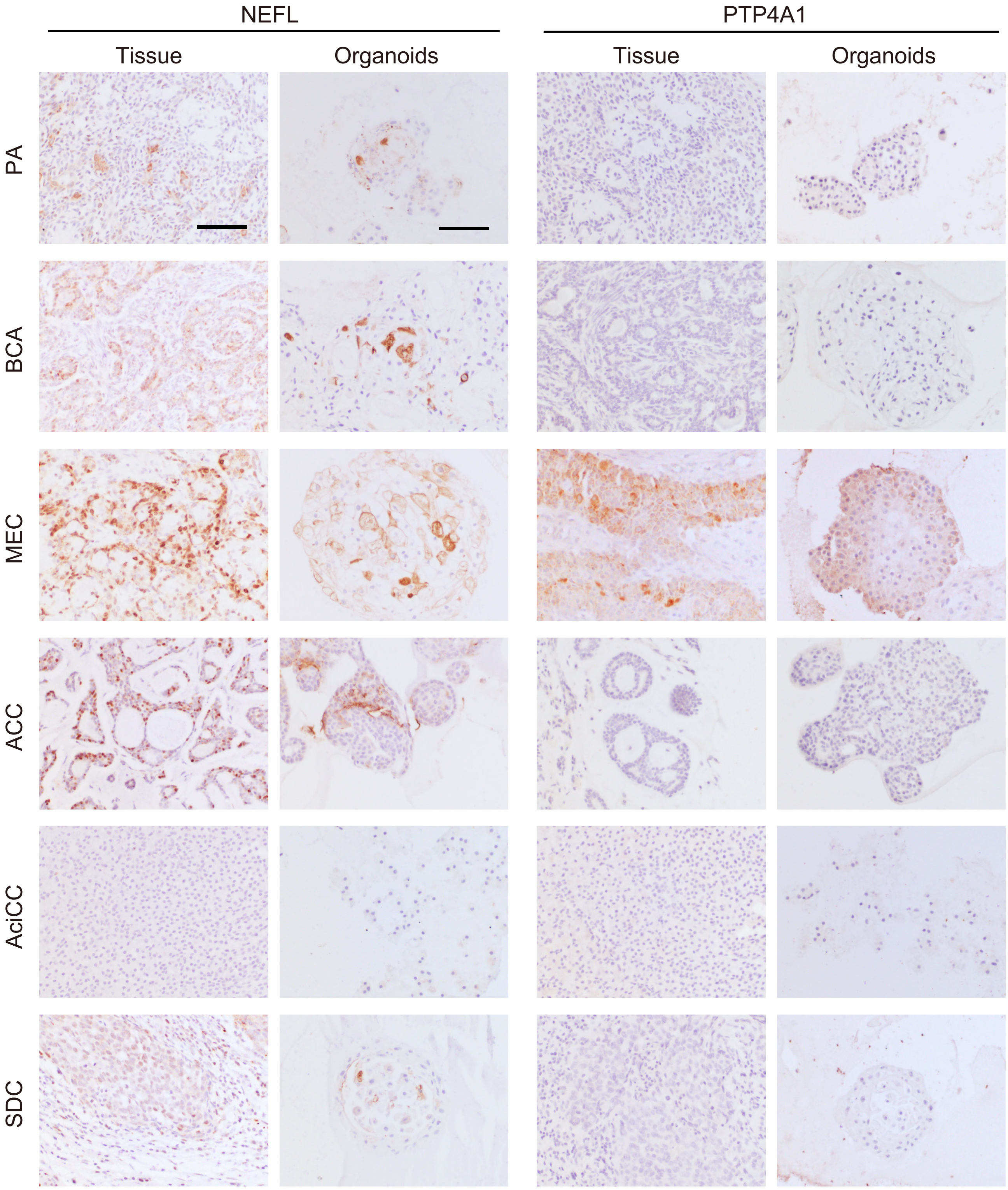

Supplementary Figure S4.
IHC staining of NEFL and PTP4A1 in SGTs organoids as compared to its parental tissues. Scale bar, 50 μm.

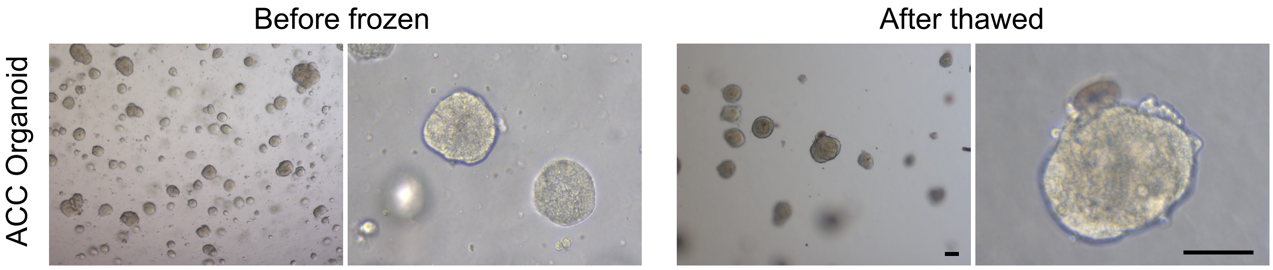

Supplementary Figure S5.
Images of ACC organoids before frozen and after thawed. Scale bar, 100 μm.
